# Supplementary material for: Anthropometric, biochemical, dietary, morbidity and well-being assessments in women and children in Indonesia, India and Senegal: a UKRI GCRF Action Against Stunting Hub protocol paper
Source: BMJ Paediatr Open. 2024 Feb 27;8(Suppl 1):e001683. doi: 10.1136/bmjpo-2022-001683 (PMC10910654; doi:10.1136/bmjpo-2022-001683)
Supplement: Supplementary data [file bmjpo-2022-001683supp001.pdf]

Supplementary Table 1: Flow chart for data collection timing

**Measurements**	**2nd trim[1]**	**3rd trim**	**Birth**	**1-2 mo[2]**	**3 mo**	**4-5 mo[2]**	**6 mo**	**9 mo**	**12 mo**	**18 mo**	**24 mo**
**Anthropometry**											
Pregnant women	IS[3]	All[4]									
Women weight only				All	All	All	All	All	All	All	All
Biological fathers			All								
Infants			All		All		All	All	All	All	All
Infants weight only				All		All					
**Blood Sample**											
Pregnant women	IS	I[5]									
Infants							All				
**Dietary Intakes**											
Pregnant women	IS	All									
Infants							All	All	All	All	All
**Ultrasound**		All									
Pregnant women											
**Breastfeeding**											
Breast milk sample					All						
Questionnaires			All	All	All	All	All				
**Morbidity[6]**			All	All	All	All	All	All	All	All	All
**Mental Health**											
Hair cortisol	IS	I			All						
GMPSS[7]	IS	I			All			All		All	
EPDS[8]	IS	I			All			All		All	
MSPSS[9]	IS	I			All			All		All	
SRQ[10]		All			All			All			

[1]–trimester; [2] – data collected each month; [3] – Indonesia and Senegal only; [4] – all three countries; [5] - India only; [6] – infant morbidity data is recorded everyday; [7] - Global Measure of Perceived Stress Scale (GMPSS); [8] - Edinburgh Postnatal Depression Scale (EPDS); [9] - Multidimensional Scale of Perceived Social Support (MSPSS), [10] Self-Reporting Questionnaire (SRQ)

Supplementary Table 2: Summary of the data that will be collected from each participant and the main indicators generated for statistical analysis.

Data collection	Main Indicators
**Anthropometry**<br><br>***Pregnant women***: Weight, height, sitting height, mid-upper-arm-circumference (MUAC) and skinfolds (triceps, biceps, sub-scapular)<br><br>***Women post-partum:*** Weight<br><br>***Biological father***: Weight, height, sitting height, MUAC and skinfolds (triceps, biceps, sub-scapular for India and Senegal) and body composition for Indonesia.<br><br>***Infant:*** Birth weight, birth length, head circumference, knee-to-heel length, weight, length, MUAC, skinfolds (triceps, sub-scapular)	***Pregnant women***: Weight gain in pregnancy [1]**(con)**, sitting height to standing height ratio**(con)** and arm muscle and fat areas **(con)**<br><br>***Maternal:*** Post-partum body-mass index (BMI) [2]**(cat)** and % under-weight/over-weight /obese **(con)**<br><br>***Paternal:*** BMI **(cat)**, % under-weight/over-weight /obese **(con)**, sitting height to standing height ratio **(con)**, and arm muscle and arm fat areas **(con)**<br><br>***Infant**:* Birth weight **(con)**, % low birth weight **(con)**, Z-scores[3] for weight-for-age **(cat)**, weight-for-length **(cat)**, length-for-age **(primary outcome-cat)**, length **(primary outcome-con)**, BMI **(cat)**, skinfolds **(con)**, arm and head circumferences **(con)**, Z-scores[3] for velocity-for-length **(primary outcome-con)**, weight and head circumference **(con)**, Z-score for weight-for-length[3] **(con), %** stunted **(con)**, % wasted **(con)** and % low arm circumference **(con).**
**Blood Sample**	

Pregnant women: Blood sample in (1) BD EDTA lavender tube (6 ml India and Senegal and 5.75 ml Indonesia); (2) BD royal blue top trace element free tube (4 ml India, 1 ml Indonesia and 5 ml Senegal); and (3) BD microtainer (120 µl India and Senegal and 250 µl Indonesia)<br><br>***Infants**:* Non-fasting blood sample in (1) BD EDTA lavender tube (2.5 ml India and Senegal and 1.5 ml Indonesia); (2) BD royal blue top trace element free tube (0.7 ml India, 1 ml Indonesia and 2.5ml Senegal); and (3) BD microtainer (20 µl India, and 40 µl Senegal)	***Pregnant women*****:** (1) Micronutrient status (RBC folate, Plasma folate**,** B vitamins, homocysteine, methylmalonic acid, choline, betaine, complete blood count, ferritin, transferrin receptor, hepcidin, retinol binding protein, retinol, amino acids, Fe, Zn, Cu, Se, Ca, Cr, Cd, Co, Pb, Hg, As, Al, and 25-dihydroxyvitamin D) **(con)**; (2) RBC-fatty acids **(con)**; (3) Gestational Diabetes with HbA1C **(cat)**; and (4) Inflammation with C-reactive protein, α1-acid glycoprotein **(con)**<br><br>***Infants***: Micronutrient status (Folate, B vitamins, methylmalonic acid, choline, betaine, complete blood count, ferritin, transferrin receptor, hepcidin, retinol binding protein, retinol, RBC-fatty acids and Folate, Fe, Zn, Cu, Se, Ca, Cr, Cd, Co, Pb, Hg, As, Al) **(con);** (2) RBC-fatty acids **(con)**; (3) Amino acids **(con)**; (4) Growth Hormone, Insulin-like growth factor-1, IGF binding protein, intestinal fatty acid binding protein **(con)**, and (5) Inflammation with C-reactive protein, α1-acid glycoprotein **(con)**
**Dietary Intakes**<br><br>***Pregnant women:*** Multiple pass 24-hour diet recall with repeat in 10% of women at each time point<br><br>***Infants:*** Multiple pass 24-hour diet recall with repeat in 10% of infants at each time point	***Pregnant women:*** Intakes of selected foods/beverages (g/d) **(con)**, energy and nutrients **(con)**, % at risk of inadequate nutrient intakes **(con)**, % energy from selected food groups and food sub-groups **(con)**<br><br>***Infants:*** Intakes of selected foods/beverages (g/d), energy and nutrients **(con)**, the nutrient density of complementary feeding diets in relation to desired levels **(con)**, % energy from selected food groups and food sub-groups **(con)**, % continued breastfed to 24-months of age **(con),** complementary feeding (introduction of solid, semi-solid

	or soft foods 6–8 months, minimum dietary diversity 6–23 months, minimum meal frequency 6–23 months, minimum milk feeding frequency for non-breastfed children 6–23 months, minimum acceptable diet 6–23 months, egg and/or flesh food consumption 6–23 months, sweet beverage consumption 6–23 months, unhealthy food consumption 6–23 months and zero vegetable or fruit consumption 6–23 months)<br><br>***Infants ≥12-months:*** % at risk of inadequate nutrient intakes and nutrient density **(con)**
**Ultrasound**<br><br>**Pregnant woman and developing infant:** 1) Biparietal diameter, 2) occipito-frontal diameter, 3) head circumference, 4) antero-posterior abdominal diameter, 5) transverse abdominal diameter, 6) abdominal circumference (AC) and 7) femur length, 8) fetal presentation, 9) placental localization, 10) amniotic fluid volume, 11) fetal movement, 12) cardiac pulsation, 13) presence of congenital anomalies and 14) gestational age (GA)	 <br>**Pregnant woman and developing infant:** Intrauterine growth retardation (IUGR), gestational age **(con)** and fetal-weight-for-gestational age **(con)**
**Breastfeeding**	

Breast milk sample: Full expression of a morning breastmilk sample from one breast<br><br>***Questionnaire at birth***: Administered within 72 hours of birth on initial breastfeeding practices<br><br>***Questionnaire in first 6-months***: Administered once per month on breastfeeding practices	***Breastmilk sample:*** the breastmilk content of fatty acids, fat- and water-soluble vitamins, minerals and oligosaccharides **(con)**<br><br>***Questionnaire at birth***: % ever breastfed **(con)**, % early initiation of breastfeeding **(con)**, % pre-lacteal feeding **(con)**<br><br>***Questionnaire in first 6-months***: % exclusively breastfed **(con)**, % mixed milk feeding **(con)**, age of introduction of semi-solid or solid foods **(con)**
**Infant Morbidity**<br><br>***Paper based pictorial chart:*** Completed daily by caregivers on infant health status, illness, clinic visits and medication use	***Paper based pictorial chart:*** % frequency and number of days of reduced appetite, stomach pain[4], bloated stomach[4], diarrhoea, vomiting fever, cough, wheezing, nasal discharge, ear pain[5], chicken pox[5] or measles[5], health center visits and prescribed medication **(con)**
**Maternal mental health& well-being**<br><br>***Hair samples:*** Collected from the posterior vertex of the head from pregnant women ***Questionnaires***: Administered to the woman during pregnancy and post-partum	Hair cortisol content as an indicator of stress (>182 pg/mg) **(con)**

	Global Measure of Perceived Stress Scale (GMPSS) (0-13=low perceived stress, 14-26=moderate perceived stress and 27-40=high perceived stress) **(cat)**<br><br>Edinburgh Postnatal Depression Scale (EPDS) cut-off of 13 **(cat)**<br><br>Multidimensional Scale of Perceived Social Support (MSPSS) (12-35 indicates low perceived support, 36-60 indicates medium perceived support and 61-84 indicates high perceived support) **(cat)**

[1]continuous **(con)**; [2]categorical **(cat)**

[3]Z-scores will be calculated utilizing the WHO child growth standards software (12), [4]Senegal only, [5]India and Indonesia only,
