# Supplementary material for: Anthropometric, biochemical, dietary, morbidity and well-being assessments in women and children in Indonesia, India and Senegal: a UKRI GCRF Action Against Stunting Hub protocol paper
Source: BMJ Paediatr Open. 2024 Feb 27;8(Suppl 1):e001683. doi: 10.1136/bmjpo-2022-001683 (PMC10910654; doi:10.1136/bmjpo-2022-001683)
Supplement: Author's manuscript [file bmjpo-2022-001683.draft_revisions.pdf]

BMJ Paediatrics Open

BMJ Paediatrics Open is committed to open peer review. As part of this commitment we make the peer review history of every article we publish publicly available.

When an article is published we post the peer reviewers' comments and the authors' responses online. We also post the versions of the paper that were used during peer review. These are the versions that the peer review comments apply to.

The versions of the paper that follow are the versions that were submitted during the peer review process. They are not the versions of record or the final published versions. They should not be cited or distributed as the published version of this manuscript.

BMJ Paediatrics Open is an open access journal and the full, final, typeset and author-corrected version of record of the manuscript is available on our site with no access controls, subscription charges or pay-per-view fees (http://bmjpaedsopen.bmj.com).

If you have any questions on BMJ Paediatrics Open's open peer review process please email info.bmjpo@bmj.com

BMJ Paediatrics Open

Anthropometric, biochemical, dietary, morbidity and wellbeing assessments in women and children in Indonesia, India and Senegal: a UKRI GCRF Action Against Stunting Hub protocol paper

Journal:	*BMJ Paediatrics Open*
Manuscript ID	bmjpo-2022-001683
Article Type:	Protocol
Date Submitted by the Author:	22-Sep-2022
Complete List of Authors:	Davies-Kershaw, Hilary; LSHTM, Department of Population Health<br>Fahmida, Umi; SEAMEO RECFON<br>Htet, Min Kyaw; SEAMEO RECFON<br>Kulkarni, Bharati; National Institute of Nutrition<br>Faye, Babacar; UCAD, Service de Parasitologie-Mycologie- Pédiatrie, Faculté de médecine<br>Yanti, Dwi; SEAMEO RECFON<br>Shinta, Dewi; SEAMEO RECFON<br>Zahra, Nur ; SEAMEO RECFON<br>Angelin, Tiffany C.; SEAMEO RECFON<br>Madhari, Radhika; National Institute of Nutrition<br>Pullakhandam, Raghu; National Institute of Nutrition<br>Palika, Ravindranadh; National Institute of Nutrition<br>Fernandez Rao, Sylvia; National Institute of Nutrition<br>Selvaraj, Kiruthika; National Institute of Nutrition<br>Pratyusha, Dharani; National Institute of Nutrition<br>Kumar, Santosh; National Institute of Nutrition<br>Yadev, Dinesh; LIDC<br>Diouf, Saliou; UCAD, Service de Parasitologie-Mycologie- Pédiatrie, Faculté de médecine<br>Lopez-Sall, Philomene; UCAD, Service de Parasitologie-Mycologie- Pédiatrie, Faculté de médecine<br>Diallo, Babacar; UCAD, Service de Parasitologie-Mycologie- Pédiatrie, Faculté de médecine<br>Mouissi, Princillia; UCAD, Service de Parasitologie-Mycologie- Pédiatrie, Faculté de médecine<br>Fall, Sally; UCAD, Service de Parasitologie-Mycologie- Pédiatrie, Faculté de médecine<br>Diallo, Ibrahima; UCAD, Service de Parasitologie-Mycologie- Pédiatrie, Faculté de médecine<br>Djigal, Aicha; UCAD, Service de Parasitologie-Mycologie- Pédiatrie, Faculté de médecine<br>Immerzeel, Tabitha; LSHTM, Department of Population Health<br>Tairou, Fassia; UCAD, Service de Parasitologie-Mycologie- Pédiatrie, Faculté de médecine<br>Diop, Assana; UCAD, Service de Parasitologie-Mycologie- Pédiatrie,

	Faculté de médecine<br>Pradeilles, Rebecca; Loughborough University, School of Sport, Exercise and Health Sciences<br>Strout, Sara; LSHTM, Department of Population Health<br>Momo Kadia, Benjamin; Liverpool School of Tropical Medicine Department of Clinical Sciences,<br>Tata, Darius; LIDC<br>Jobarteh, Modou; London School of Hygiene & Tropical Medicine, Epidemiology & Population Health<br>Allen, Stephen; LSTM, Department of Clinical Sciences<br>Walker, Alan; University of Aberdeen,<br>Webster, Joanne P.; University of London, Department of Pathobiology and Population Sciences<br>Haggarty, Paul; University of Aberdeen<br>Heffernan, Claire; LIDC<br>Ferguson, Elaine; LSHTM, Department of Population Health
Keywords:	Epidemiology, Growth, Data Collection

SCHOLARONE™
Manuscripts

I, the Submitting Author has the right to grant and does grant on behalf of all authors of the Work (as defined in the below author licence), an exclusive licence and/or a non-exclusive licence for contributions from authors who are: i) UK Crown employees; ii) where BMJ has agreed a CC-BY licence shall apply, and/or iii) in accordance with the terms applicable for US Federal Government officers or employees acting as part of their official duties; on a worldwide, perpetual, irrevocable, royalty-free basis to BMJ Publishing Group Ltd ("BMJ") its licensees and where the relevant Journal is co-owned by BMJ to the co-owners of the Journal, to publish the Work in this journal and any other BMJ products and to exploit all rights, as set out in our licence.

The Submitting Author accepts and understands that any supply made under these terms is made by BMJ to the Submitting Author unless you are acting as an employee on behalf of your employer or a postgraduate student of an affiliated institution which is paying any applicable article publishing charge ("APC") for Open Access articles. Where the Submitting Author wishes to make the Work available on an Open Access basis (and intends to pay the relevant APC), the terms of reuse of such Open Access shall be governed by a Creative Commons licence – details of these licences and which Creative Commons licence will apply to this Work are set out in our licence referred to above.

Other than as permitted in any relevant BMJ Author's Self Archiving Policies, I confirm this Work has not been accepted for publication elsewhere, is not being considered for publication elsewhere and does not duplicate material already published. I confirm all authors consent to publication of this Work and authorise the granting of this licence.

Confidential: For Review Only

Anthropometric, biochemical, dietary, morbidity and wellbeing assessments in women and children in Indonesia, India and Senegal: a UKRI GCRF Action Against Stunting Hub protocol paper

Authors

Hilary Davies-Kershaw[1], Umi Fahmida[2], Min Kyaw Htet[2], Bharati Kulkarni[3], Babacar Faye[4],Dwi Yanti[2], Dewi Shinta[2], Nur Lailatuz Zahra[2], Tiffany Cornelia Angelin[2], Radhika Madhari[3], Raghu Pullakhandam[3], Ravindranadh Palika[3], Sylvia Fernandez Rao[3], Kiruthika Selvaraj[3], Dharani Pratyusha[3], Santosh Kumar[3], Dinesh Yadav[9],Saliou Diouf[4], Philomène Lopez-Sall[4], Babacar Diallo[4], Princillia Mouissi[4], Sally Fall[4], Ibrahima Diallo[4], Aicha Djigal[4], Tabitha D Van Immerzeel[1], Fassia Tairou[4], Assana Diop[4], Rebecca Pradeilles[5], Sara Strout[1], Benjamin Momo Kadia[6], Darius Tetsa, Tata[9], Modou L. Jobarteh[9], Stephen Allen[6], Alan W. Walker[7], Joanne P. Webster[8], Paul Haggarty[7], Claire Heffernan[9], Elaine L. Ferguson[1]

Authors' affiliations

[1]Department of Population Health, Faculty of Epidemiology and Population Health, London School of Hygiene and Tropical Medicine (LSHTM) United Kingdom (UK)

[2]Southeast Asian Ministers of Education Organization Regional Center for Food and Nutrition (SEAMEO RECFON), Jakarta, Indonesia

[3]ICMR - National Institute of Nutrition, Hyderabad, India

[4]Service de Parasitologie-Mycologie- Pédiatrie, Faculté de médecine, Université Cheikh Anta Diop (UCAD), Dakar, Senegal.

[5]School of Sport, Exercise and Health Sciences, Loughborough University, UK

[6]Department of Clinical Sciences, Liverpool School of Tropical Medicine (LSTM), Liverpool, UK

[7]Rowett Institute, University of Aberdeen, Aberdeen, UK

[8]Department of Pathobiology and Population Sciences, Royal Veterinary College, University of London, Hawkshead Campus, Herts, UK

[9]London International Development Centre (LIDC), London, UK

Corresponding author

Hilary Davies-Kershaw, Population Health Department, LSHTM, email: hilary.davies@lshtm.ac.uk

Confidential: For Review Only

ABSTRACT

Introduction: Child-stunting has a complex aetiology, especially in the first 1000 days of life. Nutrition interventions alone have not produced expected impacts in reducing/preventing child-stunting, indicating the importance of understanding the complex interplay between environmental, physiological and psychological factors influencing child nutritional status.

This study will investigate maternal and child nutrition, health and wellbeing status and associated factors through the assessment of:(1) anthropometry; (2) biomarkers of nutrition and health status; (3) dietary intakes; (4) fetal growth and development; (5) infant morbidity; (6) Infant and Young Child Feeding (IYCF); and (7) perinatal maternal stress, depression and social support.

Methods:

This study will be conducted in a prospective pregnancy cohort in India, Indonesia and Senegal. Pregnant women will be recruited in the second (Indonesia, Senegal) and third (India) trimester of pregnancy, and the mother and infant dyads followed until the infant is 24-months of age. During pregnancy, anthropometric measures will be taken, venous blood samples will be collected for biochemical assessment of nutrition and health status, dietary intakes will be assessed using a 4-pass-24-hour dietary recall method (MP24HR), fetal ultrasound for assessment of fetal growth. After birth, anthropometry measurements will be taken, venous blood samples will be collected, MP24HR will be conducted, infant morbidity and IYCF practices will be assessed and a sample of breastmilk will be collected for nutrient composition analyses. Perinatal maternal stress, depression, social support and hair cortisol levels (stress) will be measured. The results from this study will be integrated in an interdisciplinary analysis to examine factors influencing infant growth and inform global efforts in reducing child stunting.

Ethics and dissemination*:* Ethical approval was obtained from Institutional ethics committees in India, Indonesia, Senegal and the UK. Results will be published in peer-reviewed journals and disseminated to policy makers and participating communities.

Keywords:

Stunting, nutritional status, anthropometry, dietary intake, micronutrient status, breastmilk, infant morbidity, maternal mental health and social support

Confidential: For Review Only

SUMMARY BOX

What is known about the subject?

- The global burden of child stunting remains unacceptably high
- Whilst some progress has been made, reduction of the burden of child stunting is very slow and sometimes unresponsive
- Increasing the understanding of the complex aetiology of child stunting is critical to programmes and policies to further prevent or reduce the global burden

What this study hopes to add?

- Discern the contribution of maternal and child nutrition and nutritional status on child stunting as part of an interdisciplinary analysis
- Increase understanding of complex interrelationships and biological factors that influence maternal and child nutrition and stunting
- Determine and compare risks of undernutrition across the three settings using anthropometric, biochemical and dietary nutritional status for pregnant women and their offspring
- Contribute to an understanding of the influence of maternal depression, maternal stress and social support on child nutritional status

Confidential: For Review Only

INTRODUCTION

Adequate nutrition during the first 1000 days of life (from conception to 24-months of age) is essential for optimal linear and ponderal growth, immune function and cognitive-development(1,2). Poor maternal nutritional status during pregnancy and lactation contributes to infant undernutrition through its influence on infant birthweight and length, gestation length, infant immunity and the nutrient content of breastmilk(1,3). Important intermediate factors contributing to undernutrition after birth include the quality of infant-care and breastfeeding practices, infant's dietary intake, and the frequency and severity of infant illnesses(4). Along with the widely recognised underlying causes of undernutrition, including food-security, poverty, maternal education and health service infrastructure(4), there is a growing interest in less-researched areas such as maternal depression, stress and social support, which can influence infant-care and feeding practices(4,5). The Action Against Stunting Hub observational Study (AASH) will follow a cohort of mother-infant dyads from pregnancy to 24-months post-partum in Hyderabad, India, East Lombok, Indonesia and Kaffrine, Senegal where biological and environmental exposures and the prevalence of child-stunting differ, ranging from 26% in Kaffrine-District (Senegal), 28% in Telegana-State, (India) to 44% in East Lombok, (Indonesia)(6–8).

This study will investigate the contribution of factors affecting child growth and development through assessment of:

1) Anthropometric status of pregnant women, their infants and the infants' biological father,

2) Biochemical and dietary nutritional status of women during pregnancy and infants from 6-months of age,

3) Fetal growth and development,

4) IYCF practices and morbidity of the infants,

5) Perinatal maternal stress, depression and social support, and

6) Associations of environmental, biological and sociological factors on infant growth

Confidential: For Review Only

METHODS AND ANALYSIS

Prospective pregnancy cohort studies will be conducted in Hyderabad, India, East Lombok, Indonesia and Kaffrine, Senegal. Women will be recruited in the second (Senegal and Indonesia) or third (India- delays due to COVID-19) trimester of pregnancy. Their infants will be followed until 24-months of age. The selection of methods for the nutrition study was guided by a conceptual framework for stunted linear infant growth (Figure 1). Summaries of measurement indicators and timings of data collection are presented in Tables 1 and 2. All data, except dietary intake in India and Senegal will be recorded using a centralised data collection application (CommCare®, Dimagi, Inc., Cambridge, MA, USA).

Data collection

Anthropometric Assessment

Anthropometric measurements of growth and body composition will be conducted on pregnant women in the second and/or third trimester of pregnancy, from the infants' biological father, and on infants within 72 hours after birth and at 3, 6, 9, 12, 18 and 24-months of age by trained enumerators (Table 2). Measurements will be done in duplicate, using standardised procedures and calibrated equipment, with a third measurement taken if the first and second measurements do not agree with the pre-defined limits (Table 3)(9).

Weight will be measured using the SECA-876 scale with a mother-child-function (range=0-250kg, precision±0.1kg). Adult height (standing and sitting) and infant length will be measured using a portable UNICEF-height/length board (range=0-221cm, precision±0.1cm), and for sitting height using a purpose-built anthropometric box(10). Mid-upper arm circumference (MUAC) and infant head circumference will be measured using the Lufkin-WP601 tape-measure. Both infant and adult arm measurements will be done on the left-hand side. Knee-to-heel length will be measured using a segmometer (Malby labs-India and Indonesia and Sealy-Senegal). Skinfolds measurements will be taken using a Holtain skinfold-caliper (range 0-40mm, precision±0.2mm).

In India and Senegal, built-in consistency checks in CommCare for infant z scores; length-for-age (<-6 or >6sd), weight-for-length (<-5 or >5sd) and weight-for-length (<-6 or >5sd)(9,11), and for a negative difference between the current and preceding measurement of infant weight and length. Z-scores will be calculated using the WHO child growth standards software(12). Prior to data collection, in each country, a standardisation session will take place to calculate intra- and inter-

Confidential: For Review Only

observer absolute and relative technical error of measurements and coefficients of reliability for all anthropometric measurements (9,11). Regular refresher training sessions will be conducted and, field supervisors will monitor data collection, using a checklist based on the study's Standard Operating Procedures (SOP).

Biochemical assessment of blood samples

Using standard procedures(13), blood samples will be collected from pregnant women (i.e., 10 mL in India and Senegal and 7 mL in Indonesia) during the third trimester (and second trimester in a sub-sample of women enrolled in a concurrent egg intervention trial in Indonesia). In Infants, 3 mL of non-fasting blood samples will be collected at 6 months of age (and at 18-months in a sub-sample of children enrolled in an egg intervention study in India). The samples will be collected using Becton-Dickinson vacutainers®(EDTA and trace-element free). A complete blood count and HbA1C (women only) will be analysed on whole blood samples within 4-8 hours of collection. Blood samples will then be processed for separation of plasma, red blood cells and serum and aliquoted into microtubes for storage. All aliquots will be stored at -80°C until analyses. The time of blood sample collection and separation will be recorded. The risks of contamination/analyte deterioration will be minimised by wearing powder-free gloves, keeping samples cool and protection from light.

Blood samples will be analysed for maternal and infant biochemical indicators of nutritional status, inflammation, and growth factors in infants (Table 4). Certified reference materials (CRM) will be included in the analyses where available to ensure analytical accuracy, precision and reliability. A certified pooled plasma sample (NIST-909c) will also be analysed across all laboratories to ensure inter-country comparability and analytical reliability.

Dietary intakes

Four-pass 24-hour dietary recall (MP24HR)(14) will be used to assess dietary intake. During the second (Indonesia and Senegal) and third trimesters (all sites), women will be asked to recall all foods and beverages consumed in a preceding 24 hours. Similarly, 24-hours dietary intake assessment will be conducted during infancy at 6, 9, 12, 18 and 24-months of age to estimate infants dietary intake. Dietary intake assessment will be carried out only on a randomly selected sub-sample (i.e., 10% of participants) at each time-point. Quantities of foods and drinks consumed will be estimated by weighing actual foods/beverages or food models using calibrated digital electronic kitchen scales (Salter, 1024,-India and Senegal and Tanita, KD-160-Indonesia; precision±1.0 g), dimension measurements (cm), size estimations (small, medium or large) or using photographs, depending on the item. Details on sources of food acquisition will be recorded, and questions will be

https://mc.manuscriptcentral.com/bmjpo

Confidential: For Review Only

asked about whether their food intake on the preceding 24-hours was typical of their usual intakes, and for infants, whether they were breastfed.

Local conversion factors to convert portion size estimates to grams consumed and average recipe data to convert the quantity of a mixed-dish consumed into grams of individual ingredients will be developed when they are not available from previous studies.

In Indonesia, MP24HR data will be entered on a tablet (CommCare-application)(15), whilst in India and Senegal, a paper form will be used to collect dietary data from respondents and entered on a web-based application created specifically for this project and then uploaded on to the study data repository. Both applications (paper and tablet-format) will calculate each participant's intakes of energy and nutrients using the local conversion factors, average recipe data and country-specific food composition tables (FCT) in India and Indonesia(15,16), and a FCT developed using a hierarchy of other food composition values in Senegal(17–23). Paper-based forms will be reviewed immediately after data collection by the field supervisors and any questions resolved.

Ultrasound

Pregnant women in all three countries will have an antenatal ultrasound in government-recognised diagnostic centers at 32-weeks (± 2-weeks) to evaluate fetal growth and development(24). A trained obstetric sonographer will use a ultrasound system(25) to take the following measurements in triplicate, which will be checked by a radiologist; biparietal diameter, occipito-frontal diameter, head circumference, antero-posterior abdominal diameter, transverse abdominal diameter, abdominal circumference and femur length, fetal presentation, placental localisation and amniotic fluid volume, fetal movement, cardiac pulsation and the presence of congenital anomalies(26). Estimated gestational age will be calculated using the crown-rump length(27). The ultrasound report will be shared with the participant and advised to see the on-call doctor if necessary.

IYCF practices during the first 6 months after birth and breastmilk nutrient composition

Within 72-hours of birth, enumerators will administer a questionnaire to mothers to evaluate: 1)pre-lacteal feeding (other feeds, besides breastmilk), 2)breastfeeding initiation and timing, 3)colostrum fed to the infant, 4)other feeds, besides breastmilk, and 5)infant nutrition supplements(28,29). Once a month until the infant is 6-months of age, the enumerator will ask mothers whether their infant has consumed anything other than breastmilk during the previous four-weeks. If the infant was not exclusively breastfed, detailed questions about the type of newly introduced liquids or solids will be asked.

Confidential: For Review Only

A morning (between 6h00-12h00) breastmilk sample will be collected from mothers at 3-months post-partum by asking them to express all milk from one breast into a sterile collection bottle, using a manual breastmilk pump, following standard procedures(30). After collection the bottles will be placed in an ice-box and transported to a laboratory, where milk samples will be mixed with a vortex-mixer and aliquot 10 mL into three amber-coloured screw cap 15mL Eppendorf-tubes® (fat- and water-soluble vitamins and fatty acids analyses), one clear trace-element free tube (minerals analysis) and 1 mL in clear 1.5 mL Eppendorf-tubes (oligosaccharides analysis)(30). Samples will be immediately stored at -80°C (Table 2). CRM (NIST-1549a) – a standard control sample, will be included in the analyses to ensure analytical accuracy, precision and reliability.

Child morbidity

Child morbidity will be recorded using an adapted country-specific pictorial diary from birth to 24 months of age (31). Mothers will be asked to complete the diary daily in India and Indonesia and collected weekly. Enumerators will help complete any incomplete forms. In Senegal, morbidity data will be collected weekly via enumerator-led telephone interviews. The same information will be collected across all three countries. Mothers will be asked if her child was healthy or had active, reduced appetite, diarrhoea (with or without blood and/or mucus in the stools), vomiting, bloated stomach, fever, cough (with or without nasal discharge and/or wheezing), ear pain, chicken pox or measles(31).They will also record any health center visits and any medication the infant received. Additional morbidity questions (1-week recall) will also be asked at the same time as monthly IYCF questionnaire up to 6-months of age.

Maternal mental health and social support

Maternal stress will be assessed using cortisol level in hair samples (32) and the Global Measure of Perceived Stress Scale (GMPSS) questionnaire(33). Hair samples will be collected and GMPSS questionnaire administered during pregnancy (i.e., second trimester in Indonesia and Senegal and third trimester in India) and at 3 and 9 months post-partum. Hair grows approximately 1cm/month, therefore 3cm of hair will be collected as close as possible to the scalp from the posterior vertex of the head, placed on a hair collection card indicating the root end, wrapped in aluminum foil and stored in a dry, dark environment at room temperature until analysed, as described elsewhere(32,34). Information on hair-care practices, which influence the hair-cortisol measurements, will also be recorded(34). The GMPSS consists of 14 questions, on thoughts and feelings, which participants will rate using a 5-point Likert scale (33).

Confidential: For Review Only

Maternal depression will be measured using the Edinburgh Postnatal Depression Scale (EPDS) and the Self-Reporting Questionnaire (SRQ) during the second (Senegal and Indonesia) and third trimester (India), and then in all countries at 3, 9 and 18-months post-partum(36). The EPDS consists of 10 statements, where participants are asked to rate how they felt in the last 7-days on a four-point Likert scale(35). As well as the classic EPDS, the new shorter version of the EPDS will also be asked to validate it against the classic EPDS in the three countries(37). The SRQ was developed by the WHO to screen for common mental disorders(38). It consists of 20 questions with dichotomous yes/no answers.

Maternal social support will be assessed using the Multidimensional Scale of Perceived Social Support(39) at 3, 9 and 18-months after birth. It includes 12 questions where participants are asked to rate how strongly they agree or disagree on a 7-point Likert scale(39), and their responses are recorded.

Statistical analysis plan

Summary statistics will be reported for each exposure and co-variate (Table 2) and grouped according to the outcome of interest as depicted in the conceptual framework (figure 1). Confounders and effect modifiers will be determined *a priori*. Directed Acyclic Graphs (DAGS) will be developed to understand possible confounders, effect modifiers and mediators on the causal pathway between exposures and outcomes. The longitudinal repeated measures will be analysed using generalised estimating equations (GEE), with unstructured correlation and robust standard errors, to model and understand potential predictors of outcomes and allow for clustering. STATA (version 17) statistical software will be used.

Patient and public partnership statement

Patients and public were not involved in the design of this study

Discussion

Addressing early life undernutrition is a public health priority in LMICs given its detrimental impacts on child survival, growth and development. The full extent and aetiology of undernutrition, however, are not well established. given the limited number of cohort studies from fetal life through to early childhood. As such studies are needed to understand the complex interplay among environmental and intrinsic physiological factors influencing early life nutritional status in different contexts.

Confidential: For Review Only

This study and others within the Action Against Stunting Hub (AASH) will investigate these complex relationships across three distinct geographies in sub-Saharan Africa and Asia. The analysis will examine factors affecting infant growth through three distinct periods; fetal period, 0-6 months of age and during the complementary feeding (6-24 months of age). The selection of its data collection protocols is underpinned by a theoretical framework of factors influencing infant and childhood growth (Figure 1), which will be further expanded through AASH inter-disciplinary collaborations to include influences of epigenetics, gut-health and food-systems. This study will also contribute to less-researched areas such as the influence of maternal depression, maternal stress and social support on infant care and feeding practices which ultimately affect infant-growth.

There are several limitations to this study. Firstly, although identical indicators of micronutrient status will be analysed across countries, the methods of analyses for two biochemical indicators differ. To minimize the influence of inter-laboratory assay differences on cross-country comparisons, a pooled-sample and CRMs, where available, will be analysed in all laboratories. Secondly, inter-population differences in maternal literacy necessitated a change in morbidity data collection protocols in Senegal. Thirdly, when investigating causal inferences, there are limitations with a prospective cohort study design, including loss-to-follow-up bias and confounding. These biases will be minimised through concerted sensitisation/motivational efforts to reduce loss-to-follow-up and the use of Directed-Acylic-Graphs.

Conclusions

Results from the nutrition study conducted in three diverse geographic regions will form part of the ‘whole child approach’ pursued under AASH. The interdisciplinary collaborations established across the hub provide a unique opportunity to investigate the complex inter-relationships among environmental and intrinsic physiological factors influencing early life growth and development. Such insights are important for informing effective intervention design and the policy actions needed to support global efforts in reducing early child-stunting.

Confidential: For Review Only

Figure 1: A conceptual framework of pathways to assess the impact of nutrition, nutritional status and maternal wellbeing on child stunting

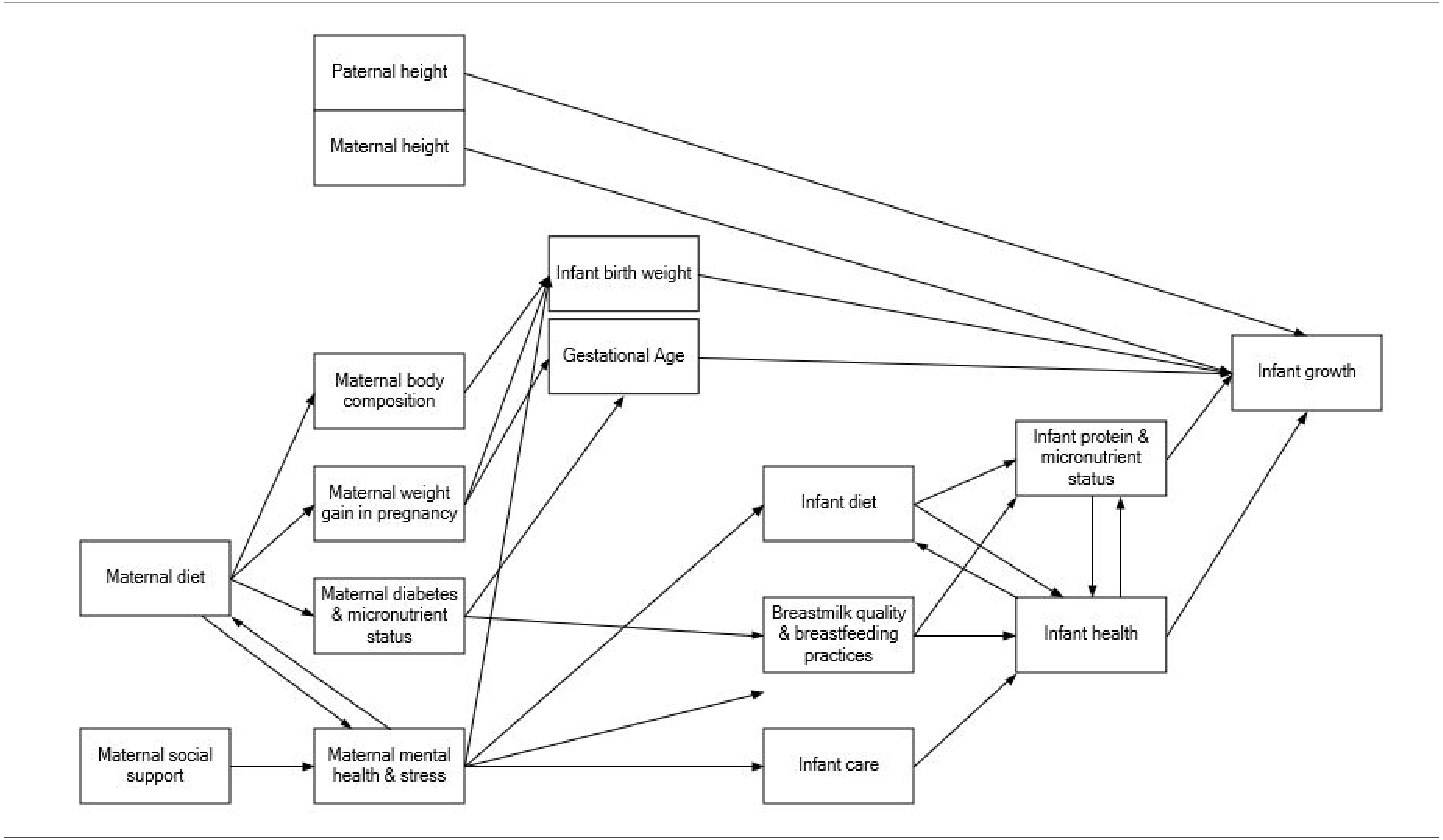

Table 1: Flow chart for data collection timing

Measurements	2nd trim[1]	3rd trim	Birth	1-2 mo[2]	3 mo	4-5 mo[2]	6 mo	9 mo	12 mo	18 mo	24 mo
**Anthropometry**											
Pregnant women	IS[3]	All[4]									
Women weight only				All	All	All	All	All	All	All	All
Biological fathers			All								
Infants			All		All		All	All	All	All	All
Infants weight only				All		All					
**Blood Sample**											
Pregnant women	IS	I[5]									
Infants							All				
**Dietary Intakes**											
Pregnant women	IS	All									
Infants							All	All	All	All	All
**Ultrasound**		All									
Pregnant women											
**Breastfeeding**											
Breast milk sample					All						
Questionnaires			All	All	All	All	All				
**Morbidity[6]**			All	All	All	All	All	All	All	All	All
**Mental Health**											
Hair cortisol	IS	I			All						
GMPSS[7]	IS	I			All			All		All	
EPDS[8]	IS	I			All			All		All	
MSPSS[9]	IS	I			All			All		All	
SRQ[10]		All			All			All			

[1]–trimester; [2] – data collected each month; [3] – Indonesia and Senegal only; [4] – all three countries; [5] - India only; [6] – infant morbidity data is recorded everyday; [7] - Global Measure of Perceived Stress Scale (GMPSS); [8] - Edinburgh Postnatal Depression Scale (EPDS); [9] - Multidimensional Scale of Perceived Social Support (MSPSS), [10] Self-Reporting Questionnaire (SRQ)

Confidential: For Review Only

Table 2: Summary of the data that will be collected from each participant and the main indicators generated for statistical analysis.

Data collection	Main Indicators for statistical analysis
**Anthropometry**	
Pregnant women: Weight, height, sitting height, mid-upper-arm-circumference (MUAC) and skinfolds (triceps, biceps, sub-scapular)	***Pregnant women***: Weight gain in pregnancy, sitting height to standing height ratio and arm muscle and fat areas
Women post-partum: Weight	***Maternal:*** Post-partum body-mass index (BMI) and % under-weight/over-weight /obese
Biological father: Weight, height, sitting height, MUAC and skinfolds (triceps, biceps, sub-scapular)	***Paternal:*** BMI, % under-weight/over-weight /obese, sitting height to standing height ratio, and arm muscle and arm fat areas
Infant: Birth weight, birth length, head circumference, knee-to-heel length, weight, length, MUAC, skinfolds (triceps, sub-scapular)	***Infant****:* Birth weight, % low birth weight, Z-scores[1] for weight-for-age, weight-for-length length-for-age, length, BMI, skinfolds, arm and head circumferences, Z-scores[1] for velocity-for-length, weight and head circumference, Z-score for weight-for-length[1], % stunted, % wasted and % low arm circumference.
**Blood Sample**	
Pregnant women: Blood sample in (1) BD EDTA lavender tube (6 ml India and Senegal and 5.75 ml Indonesia); (2) BD royal blue top trace element free tube (4 ml India, 1 ml Indonesia and 5 ml Senegal); and (3) BD microtainer (120 µl India and Senegal and 250 µl Indonesia)	***Pregnant women***: (1) Micronutrient status (RBC folate, Plasma folate**,** B12, homocysteine, methylmalonic acid, choline, betaine, complete blood count, ferritin, transferrin receptor, hepcidin, retinol binding protein, retinol, amino acids, Fe, Zn, Cu, Se, Ca, Cr, Cd, Co, Pb, Hg, As, Al, and 25-dihydroxyvitamin D; (2)RBC-fatty acids; (3) Gestational Diabetes with HbA1C; and (4)Inflammation with C-reactive protein, α1-acid glycoprotein
Infants*:* Non-fasting blood sample in (1) BD EDTA lavender tube (2.5 ml India and Senegal and 1.5 ml Indonesia); (2) BD royal blue top trace element free tube (0.7 ml India, 1 ml Indonesia and 2.5ml Senegal); and (3) BD microtainer (20 µl India, and 40 µl Senegal)	***Infants***: Micronutrient status (Folate, B_{12}, methylmalonic acid, choline, betaine, complete blood count, ferritin, transferrin receptor, hepcidin, retinol binding protein, retinol, RBC-fatty acids and Folate, Fe, Zn, Cu, Se, Ca, Cr, Cd, Co, Pb, Hg, As, Al); **(2)** RBC-fatty acids; (3) Amino acids; (4) Growth Hormone, Insulin-like growth factor-1, IGF binding protein, intestinal fatty acid binding protein, and (5) Inflammation with C-reactive protein, α1-acid glycoprotein

Confidential: For Review Only

**Dietary Intakes**	
Pregnant women: Multiple pass 24-hour diet recall with repeat in 10% of women at each time point	***Pregnant women:*** Intakes of selected foods/beverages (g/d), energy and nutrients, % at risk of inadequate nutrient intakes, % energy from selected food groups and food sub-groups
Infants: Multiple pass 24-hour diet recall with repeat in 10% of infants at each time point	***Infants:*** Intakes of selected foods/beverages (g/d), energy and nutrients, the nutrient density of complementary feeding diets in relation to desired levels, % energy from selected food groups and food sub-groups, % continued breastfed to 24-months of age<br>***Infants ≥12-months:*** % at risk of inadequate nutrient intakes and nutrient density
**Ultrasound**	
**Pregnant woman and developing infant:** 1) Biparietal diameter, 2) occipito-frontal diameter, 3) head circumference, 4) antero-posterior abdominal diameter, 5) transverse abdominal diameter, 6) abdominal circumference (AC) and 7) femur length, 8) fetal presentation, 9) placental localization, 10) amniotic fluid volume, 11) fetal movement, 12) cardiac pulsation, 13) presence of congenital anomalies and 14) gestational age (GA)	**Pregnant woman and developing infant:** Intrauterine growth retardation (IUGR), gestational age and fetal-weight-for-gestational age
**Breastfeeding**	
Breast milk sample: Full expression of a morning breastmilk sample from one breast	***Breastmilk sample:*** the breastmilk content of fatty acids, fat- and water-soluble vitamins, minerals and oligosaccharides
Questionnaire at birth: Administered within 72 hours of birth on initial breastfeeding practices	***Questionnaire at birth***: % ever breastfed, % early initiation of breastfeeding, % pre-lacteal feeding,
Questionnaire in first 6-months: Administered once per month on breastfeeding practices	***Questionnaire in first 6-months***: % exclusively breastfed, % mixed milk feeding, age of introduction of semi-solid or solid foods

Confidential: For Review Only

**Infant Morbidity**<br>***Paper based pictorial chart:*** Completed daily by caregivers on infant health status, illness, clinic visits and medication use	***Paper based pictorial chart:*** % frequency and number of days of reduced appetite, stomach pain[2], bloated stomach[2], diarrhoea, vomiting fever, cough, wheezing, nasal discharge, ear pain[3], chicken pox[3] or measles[3], health center visits and prescribed medication
**Maternal mental health& well-being**<br>***Hair samples:*** Collected from the posterior vertex of the head from pregnant women ***Questionnaires***: Administered to the woman during pregnancy and post-partum	Hair cortisol content as an indicator of stress (>182 pg/mg)<br><br>Global Measure of Perceived Stress Scale (GMPSS) (0-13=low perceived stress, 14-26=moderate perceived stress and 27-40=high perceived stress)<br>Edinburgh Postnatal Depression Scale (EPDS) cut-off of 13<br>Multidimensional Scale of Perceived Social Support (MSPSS) (12-35 indicates low perceived support, 36-60 indicates medium perceived support and 61-84 indicates high perceived support)

[1]Z-scores will be calculated utilizing the WHO child growth standards software (12), [2]Senegal only, [3] India and Indonesia only,

Confidential: For Review Only

Table 3: Pre-defined limits in the difference between first and second anthropometry measurements

Measure	Pre-defined limits
Adult and infant weight	0.1 kg
Adult height	0.5 cm
Adult Mid Upper Arm Circumference	0.5 cm
Adult skinfolds	0.2 cm/2 mm
Infant length	0.7 cm
Infant Head circumference	0.5 cm
Infant knee to heel	0.5 cm
Infant Mid Upper Arm Circumference	0.5 cm
Infant skinfolds	0.2 cm/2 mm

Confidential: For Review Only

Table 4: Analysis methods for parameters in India, Indonesia and Senegal

Parameters	India	Indonesia	Senegal
**Blood samples**			
Complete Blood Count<br>Glycated Haemaglobin (HbA1C -pregnant women only)	CBC auto-analyser (Horiba ABX Micros ES60)<br>HbA1C analyser (Affinion ™ Hb1Ac, Abbot)	CBC auto-analyser (SYSMEX XP100)<br>HbA1C analyser (PocketChem A1c, Arkray Inc)	CBC auto-analyser<br>HbA1C analyser (HPLC D10 Biorad)
Red Blood Cell folate<br>Vitamin D<br>Vitamin B12<br>Vitamin B9<br>Methylmalonic acid (MMA)<br>Choline<br>Betaine	LC-MS/MS[1]	LC-MS/MS	LC-MS/MS
Homocysteine	LC-MS/MS	HPLC[2]	HPLC
Red Blood Cell-Fatty acids	GC-FID[3]	GC-FID	GC-FID
Amino Acids	HPLC	LC-MS/MS	LC-MS/MS
Retinol	HPLC	HPLC	HPLC
Ferritin<br>TfR<br>C-reactive protein<br>α1-Acid glycoprotein<br>Hepcidin<br>Retinol binding protein<br>Insulin like growth factor-1<br>Insulin-like growth factor binding protein<br>Intestinal fatty-acid binding protein	ELISA[4]	ELISA	ELISA
Minerals/trace elements	ICP-MS[5]	ICP-MS	ICP-MS
Remaining Red blood cells<br>Remaining plasma	Kept at -80°C	Kept at -80°C	Kept at -80°C
**Breastmilk samples**			
Fat-soluble vitamins A and D	HPLC/LC-MS[6]	HPLC/LC-MS	HPLC/LC-MS
Water-soluble vitamins B1, B2, B3, B6, B9 and B12	LC-MS[7]	LC-MS	LC-MS
Fatty acids	GC-FID	GC-FID	GC-FID
Minerals	ICP-MS	ICP-MS	ICP-MS
Oligosaccharides	SPEC[8] and HPLC	SPEC and HPLC	SPEC and HPLC
Remaining breastmilk	Kept at -80°C	Kept at -80°C	Kept at -80°C

Confidential: For Review Only

**Hair samples**			
Cortisol	ELISA	ELISA	ELISA

[1] Liquid Chromatography with tandem Mass Spectrometry (LC-MS/MS), [2] High Performance Liquid Chromatography, [3] Gas Chromatography-Flame-ionisation detection (GC-FID),[4] Enzyme-Linked Immunoassay, [5] Inductively coupled plasma mass spectrometry, [6] High Performance Liquid Chromatography/ Liquid Chromatography-Mass Spectrometry [7]Liquid Chromatography-Mass Spectrometry, [8] Solid Phase Extraction Chromatography

Confidential: For Review Only

References

1. Lee RD, Nieman DC. Nutritional Assessment. International Edition. In: Forth Edition. New York: McGraw-Hill; 2007.

2. Zoleko-Manego R, Mischlinger J, Dejon-Agobé JC, *et al*. Birth weight, growth, nutritional status and mortality of infants from Lambaréné and Fougamou in Gabon in their first year of life. *PLOS ONE*. 2021;16(2).

3. Woldeamanuel GG, Geta TG, Mohammed TP, *et al*. Effect of nutritional status of pregnant women on birth weight of newborns at Butajira Referral Hospital, Butajira, Ethiopia. *SAGE Open Med*. 2019;7.

4. Black R, Allen L, Bhutta Z, *et al*. Maternal and child undernutrition: global and regional exposures and health consequences. *Lancet*. 2008;371(9608):243–60.

5. UNICEF. Nutrition, for every child UNICEF Nutrition Strategy 2020-2030. UNICEF Nutrition Section, Programme Division; 2020.

6. National Agency for Statistics and Demography - ANSD / Senegal and ICF. Demographic and Continuous Health Survey (EDS-Continue) 2017 [Internet]. Dakar, Senegal: ANSD and ICF; 2018. Available from: http://dhsprogram.com/pubs/pdf/FR345/FR345.pdf.

7. International Institute for Population Sciences. National Family Health Survey NFHS-4, 2015-16. India, Mumbai:IIPS; 2017.

8. Agency of Health Research and Development. Indonesia Basic Health Research 2018 . Indonesia; 2018.

9. De Onis M, Onyango AW, Van den Broeck J, *et al*. Measurement and standardization protocols for anthropometry used in the construction of a new international growth reference. *Food Nutr Bullitin*. 2004;25(S1):S27–36.

10. Norton K. International Standards for Anthropometric Assessment. International Society for the Advancement of Kinanthropometry; 2001.

11. WHO. Recommendations for data collection, analysis and reporting on anthropometric indicators in children under 5 years old. Geneva: World Health Organization and the United Nations Chhildren's Fund (UNICEF); 2019. Report No.: Licence: CC BY-NC-SA 3.0 IGO.

12. WHO: Geneva. WHO Anthro for personal computers, version 3.2.2, 2011: Software for assessing growth and development of the world's children. 2010. Available from: (http://www.who.int/childgrowth/software/en/

13. Centre for Disease Control and Prevention. National Health and Nutrition Examination Survey (NHANES) MEC Laboratory Procedures Manual. 2020.

14. Gibson RS, Ferguson EL. An Interactive 24-Hour Recall for Assessing the Adequacy of Iron and Zinc Intakes in Developing Countrie. Washington, DC, USA: International Life Sciences Institute; 2008.

15. Htet MK, Fahmida U, Do TT, *et al*. The used of tablet-based multiple-pass 24-hour dietary recall application (MP24Diet) to collect intake of children under two years old in the prospective cohort study in Indonesia. *Nutrients*. 2019;11(12):2889.

16. Data Komposisi Pangan Indonesia (Indonesian Food Composition Table). https://www.panganku.org/id-ID/beranda. 2018.

17. Longvah T, Anathan R, Bhaskarachary K, *et al*. Indian Food Composition Tables. National Institute of Nutrition, Indian Council of Medical Research, Department of Health Research, Hyderabad, India; 2017.

Confidential: For Review Only

18. FAO/INFOODS. Food Composition Table for Western Africa (2019) /Table de composition des aliments FAO/INFOODS pour l'Afrique de l'Ouest. 2019.

19. Nigeria Food Database. Available from: http://nigeriafooddata.ui.edu.ng/

20. Prynne C, Paul A. Food Composition Table for Use in The Gambia. Cambridge: Medial Research Council Human Nutrition Research; 2011.

21. SAFOODS, SA MRC. South African Food Data System. 2018. Available from: http://safoods.mrc.ac.za/

22. Public Health England. McCance and Widdowson's The Composition of Foods Integrated Dataset 2021. 2021. Report No.: GW-2010.

23. US Department of Agriculture. Food and Nutrition Database. Available from: https://fdc.nal.usda.gov/

24. Salomon L, Alfirevic Z, Da Silva-Costa F. ISUOG Practice Guidelines: ultrasound assessment of fetal biometry and growth. *Ultrasound Obstet Gynecol*. 2015;46(4):391–7.

25. Phillips Affiniti 50 Ultrasound system. Available from: https://www.philips.co.in/healthcare/product/HC795208/affiniti-50-ultrasound-system

26. Romosan G, Henriksson E, Rylander A, *et al*. Diagnostic performance of routine ultrasound screening for fetal abnormalities in an unselected Swedish population in 2000–2005. *Ultrasound Obstet Gynecol*. 2009;34(5):526–33.

27. Gadsbøll, K, Wright A, Kristensen SE, *et al*. Crown–rump length measurement errors: impact on assessment of growth. *Ultrasound Obstet Gynecol*. 2021;58(3):354–9.

28. Caulfield LE, Bose A, Chandyo RK,*et al*. Infant Feeding Practices, Dietary Adequacy, and Micronutrient Status Measures in the MAL-ED Study. *Clin Infect Dis.* 2014;59 Suppl 4:S248-54.

29. WHO. Indicators for assessing infant and young child feeding practices. Part 2 Measurement. Geneva, Switzerland; 2010.

30. Leghi GE, Middleton PF, Netting MJ, *et al*. A Systematic Review of Collection and Analysis of Human Milk for Macronutrient Composition. *Methodol Math Model.* 2020;00:1–19.

31. Richard SA, Barrett L, Guerrant R. Disease surveillance methods used in the 8-stie MAL-ED cohort study. *Clin Infect Dis*. 2014;59(Suppl 4):S220–4.

32. Greef M, Levine J, Abuzgaia A, *et al* . Hair cortisol analysis: An update on methodological considerations and clinical applications. *Clin Biochem*. 2019;63:1–9.

33. Cohen S, Kamarck T, Mermelstein R. A global measure of perceived stress. *J Health Soc Behav*. 1983;24:385–96.

34. Wright K, Ford J, Perazzo J, *et al*. Collecting Hair Samples for Hair Cortisol Analysis in African Americans. *J Vis Exp.* 2018;136(e57288):1–7.

35. Cox JL, Holden JM, Segovsky R. Edinburgh Postnatal Depression Scale (EPDS). *Br J Psychiatry.* 1987;150.

36. Harding T, DeArango V, Baltazar J. Mental disorders in primary health care: a study of their frequency and diagnosis in four developing countries. *Psychol Med.* 1980;10:231–41.

37. Van Heyningen T, Myer L, Tomlinson M, *et al*. The development of an ultra-short, maternal mental health screening tool in South Africa. *Glob Ment Health*. 2019;6(e24):1–14.

38. Beusenberg M, Orley JH. A User's guide to the self reporting questionnaire: A User's guide to the self reporting questionnaire. Geneva: World Health Organisation. Division of Mental Health; 1984. Available from: https://apps.who.int/iris/handle/10665/61113

Confidential: For Review Only

39. Zimet GD, Dahlem NW, Zimet SG, *et al*. The Multidimensional Scale of Perceived Social Support. *J Pers Assess.* 1988;52:30–41.

40. Cetina L, Bühlingb K, Demirc K, *et al*. Impact of Micronutrient Status during Pregnancy on Early Nutrition Programming. *Ann Nutr Metab*. 2019;74:269–78.

Confidential: For Review Only

Authors' contributions: HDK and ELF were responsible for the overall design, training and overseeing implementation of the research. UF, MKH, BK, BF, RM, RP, RP, SF, SD, RP, SA, AW, JW, PH, CH were involved in its design. UF, MKH, BK, BF, DY, DS, NLZ, TCA, RM, RP, RP, SFR, KS, DP, SK, DY, SD, PL-S, BD, PM, SF, ID, AD, TVI, FT, AD, SS, BMK, DTT implemented the research. HD-K and ELF wrote the manuscript. All authors read, provided comments on and approved the final version of the manuscript.

Funding: This study is funded by UKRI Global Challenges Research Fund (GCRF) (No. MR/S01313X/1). The views expressed in this work are those of the authors and not the funder.

Competing interest statement: The authors declare no conflict of interest.

Confidential: For Review Only

BMJ Paediatrics Open

Anthropometric, biochemical, dietary, morbidity and wellbeing assessments in women and children in Indonesia, India and Senegal: a UKRI GCRF Action Against Stunting Hub protocol paper

Journal:	*BMJ Paediatrics Open*
Manuscript ID	bmjpo-2022-001683.R1
Article Type:	Protocol
Date Submitted by the Author:	07-Dec-2022
Complete List of Authors:	Davies-Kershaw, Hilary; LSHTM, Department of Population Health<br>Fahmida, Umi; SEAMEO RECFON<br>Htet, Min Kyaw; SEAMEO RECFON<br>Kulkarni, Bharati; National Institute of Nutrition<br>Faye, Babacar; UCAD, Service de Parasitologie-Mycologie- Pédiatrie, Faculté de médecine<br>Yanti, Dwi; SEAMEO RECFON<br>Shinta, Dewi; SEAMEO RECFON<br>Zahra, Nur ; SEAMEO RECFON<br>Angelin, Tiffany C.; SEAMEO RECFON<br>Madhari, Radhika; National Institute of Nutrition<br>Pullakhandam, Raghu; National Institute of Nutrition<br>Palika, Ravindranadh; National Institute of Nutrition<br>Fernandez Rao, Sylvia; National Institute of Nutrition<br>Selvaraj, Kiruthika; National Institute of Nutrition<br>Palepu, Dharani; National Institute of Nutrition<br>B, Santosh Kumar; National Institute of Nutrition<br>Yadev, Dinesh; LIDC<br>Diouf, Saliou; UCAD, Service de Parasitologie-Mycologie- Pédiatrie, Faculté de médecine<br>Lopez-Sall, Philomene; UCAD, Service de Parasitologie-Mycologie-Pédiatrie, Faculté de médecine<br>Diallo, Babacar; UCAD, Service de Parasitologie-Mycologie- Pédiatrie, Faculté de médecine<br>Mouissi, Princillia; UCAD, Service de Parasitologie-Mycologie- Pédiatrie, Faculté de médecine<br>Fall, Sally; UCAD, Service de Parasitologie-Mycologie- Pédiatrie, Faculté de médecine<br>Diallo, Ibrahima; UCAD, Service de Parasitologie-Mycologie- Pédiatrie, Faculté de médecine<br>Djigal, Aicha; UCAD, Service de Parasitologie-Mycologie- Pédiatrie, Faculté de médecine<br>Immerzeel, Tabitha; LSHTM, Department of Population Health<br>Tairou, Fassia; UCAD, Service de Parasitologie-Mycologie- Pédiatrie, Faculté de médecine<br>Diop, Assana; UCAD, Service de Parasitologie-Mycologie- Pédiatrie,

Faculté de médecine
Pradeilles, Rebecca; Loughborough University, School of Sport, Exercise and Health Sciences
Strout, Sara; LSHTM, Department of Population Health
Momo Kadia, Benjamin; Liverpool School of Tropical Medicine Department of Clinical Sciences,
Tata, Darius; LIDC
Jobarteh, Modou; London School of Hygiene & Tropical Medicine, Epidemiology & Population Health
Allen, Stephen; LSTM, Department of Clinical Sciences
Walker, Alan; University of Aberdeen,
Webster, Joanne P.; University of London, Department of Pathobiology and Population Sciences
Haggarty, Paul; University of Aberdeen
Heffernan, Claire; LIDC
Ferguson, Elaine; LSHTM, Department of Population Health

Keywords: Epidemiology, Growth, Data Collection

SCHOLARONE™
Manuscripts

I, the Submitting Author has the right to grant and does grant on behalf of all authors of the Work (as defined in the below author licence), an exclusive licence and/or a non-exclusive licence for contributions from authors who are: i) UK Crown employees; ii) where BMJ has agreed a CC-BY licence shall apply, and/or iii) in accordance with the terms applicable for US Federal Government officers or employees acting as part of their official duties; on a worldwide, perpetual, irrevocable, royalty-free basis to BMJ Publishing Group Ltd ("BMJ") its licensees and where the relevant Journal is co-owned by BMJ to the co-owners of the Journal, to publish the Work in this journal and any other BMJ products and to exploit all rights, as set out in our licence.

The Submitting Author accepts and understands that any supply made under these terms is made by BMJ to the Submitting Author unless you are acting as an employee on behalf of your employer or a postgraduate student of an affiliated institution which is paying any applicable article publishing charge ("APC") for Open Access articles. Where the Submitting Author wishes to make the Work available on an Open Access basis (and intends to pay the relevant APC), the terms of reuse of such Open Access shall be governed by a Creative Commons licence – details of these licences and which Creative Commons licence will apply to this Work are set out in our licence referred to above.

Other than as permitted in any relevant BMJ Author's Self Archiving Policies, I confirm this Work has not been accepted for publication elsewhere, is not being considered for publication elsewhere and does not duplicate material already published. I confirm all authors consent to publication of this Work and authorise the granting of this licence.

Confidential: For Review Only

Anthropometric, biochemical, dietary, morbidity and wellbeing assessments in women and children in Indonesia, India and Senegal: a UKRI GCRF Action Against Stunting Hub protocol paper

Authors

Hilary Davies-Kershaw[1], Umi Fahmida[2], Min Kyaw Htet[2], Bharati Kulkarni[3], Babacar Faye[4],Dwi Yanti[2], Dewi Shinta[2], Nur Lailatuz Zahra[2], Tiffany Cornelia Angelin[2], Radhika Madhari[3], Raghu Pullakhandam[3], Ravindranadh Palika[3], Teena Rajeev[3] ,Sylvia Fernandez Rao[3], Santosh Kumar[3] ,Kiruthika Selvaraj[3], Dharani Pratyusha[3], Dinesh Yadav[3,9],Saliou Diouf[4], Philomène Lopez-Sall[4], Babacar Diallo[4], Princillia Mouissi[4], Sally Fall[4], Ibrahima Diallo[4], Aicha Djigal[4], Tabitha D Van Immerzeel[1], Fassia Tairou[4], Assana Diop[4], Rebecca Pradeilles[5], Sara Strout[1], Benjamin Momo Kadia[6], Darius Tetsa, Tata[9], Modou L. Jobarteh[9], Stephen Allen[6], Alan W. Walker[7], Joanne P. Webster[8], Paul Haggarty[7], Claire Heffernan[9], Elaine L. Ferguson[1]

Authors' affiliations

[1]Department of Population Health, Faculty of Epidemiology and Population Health, London School of Hygiene and Tropical Medicine (LSHTM) United Kingdom (UK)

[2]Southeast Asian Ministers of Education Organization Regional Center for Food and Nutrition (SEAMEO RECFON), Jakarta, Indonesia

[3]ICMR - National Institute of Nutrition, Hyderabad, India

[4]Service de Parasitologie-Mycologie- Pédiatrie, Faculté de médecine, Université Cheikh Anta Diop (UCAD), Dakar, Senegal.

[5]School of Sport, Exercise and Health Sciences, Loughborough University, UK

[6]Department of Clinical Sciences, Liverpool School of Tropical Medicine (LSTM), Liverpool, UK

[7]Rowett Institute, University of Aberdeen, Aberdeen, UK

[8]Department of Pathobiology and Population Sciences, Royal Veterinary College, University of London, Hawkshead Campus, Herts, UK

[9]London International Development Centre (LIDC), London, UK

Corresponding author

Hilary Davies-Kershaw, Population Health Department, LSHTM, email: hilary.davies@lshtm.ac.uk

Confidential: For Review Only

ABSTRACT

Introduction: Child-stunting has a complex aetiology, especially in the first 1000 days of life. Nutrition interventions alone have not produced expected impacts in reducing/preventing child-stunting, indicating the importance of understanding the complex interplay between environmental, physiological and psychological factors influencing child nutritional status.

This study will investigate maternal and child nutrition, health and wellbeing status and associated factors through the assessment of:(1) anthropometry; (2) biomarkers of nutrition and health status; (3) dietary intakes; (4) fetal growth and development; (5) infant morbidity; (6) Infant and Young Child Feeding (IYCF); and (7) perinatal maternal stress, depression and social support.

Methods:

This study will be conducted in a prospective pregnancy cohort in India, Indonesia and Senegal. Pregnant women will be recruited in the second (Indonesia, Senegal) and third (India) trimester of pregnancy, and the mother and infant dyads followed until the infant is 24-months of age. During pregnancy, anthropometric measures will be taken, venous blood samples will be collected for biochemical assessment of nutrition and health status, dietary intakes will be assessed using a 4-pass-24-hour dietary recall method (MP24HR), fetal ultrasound for assessment of fetal growth. After birth, anthropometry measurements will be taken, venous blood samples will be collected, MP24HR will be conducted, infant morbidity and IYCF practices will be assessed and a sample of breastmilk will be collected for nutrient composition analyses. Perinatal maternal stress, depression, social support and hair cortisol levels (stress) will be measured. The results from this study will be integrated in an interdisciplinary analysis to examine factors influencing infant growth and inform global efforts in reducing child stunting.

Ethics and dissemination*:* Ethical approval was obtained from Institutional ethics committees in India, Indonesia, Senegal and the UK. Results will be published in peer-reviewed journals and disseminated to policy makers and participating communities.

Keywords:

Stunting, nutritional status, anthropometry, dietary intake, micronutrient status, breastmilk, infant morbidity, maternal mental health and social support

Confidential: For Review Only

1 **SUMMARY BOX**

What is known about the subject?

- The global burden of child stunting remains unacceptably high
- Whilst some progress has been made, reduction of the burden of child stunting is very slow and sometimes unresponsive
- Increasing the understanding of the complex aetiology of child stunting is critical to programmes and policies to further prevent or reduce the global burden

What this study hopes to add?

- Discern the contribution of maternal and child nutrition and nutritional status on child stunting as part of an interdisciplinary analysis
- Increase understanding of complex interrelationships and biological factors that influence maternal and child nutrition and stunting
- Determine and compare risks of undernutrition across the three settings using anthropometric, biochemical and dietary nutritional status for pregnant women and their offspring
- Contribute to an understanding of the influence of maternal depression, maternal stress and social support on child nutritional status

2

3

Confidential: For Review Only

INTRODUCTION

Adequate nutrition during the first 1000 days of life (from conception to 24-months of age) is essential for optimal linear and ponderal growth, immune function and cognitive-development(1,2). Poor maternal nutritional status during pregnancy and lactation contributes to infant undernutrition through its influence on infant birthweight and length, gestation length, infant immunity and the nutrient content of breastmilk(1,3). Important intermediate factors contributing to undernutrition after birth include the quality of infant-care and breastfeeding practices, infant's dietary intake, and the frequency and severity of infant illnesses(4). Along with the widely recognised underlying causes of undernutrition, including food-security, poverty, maternal education and health service infrastructure(4), there is a growing interest in less-researched areas such as maternal depression, stress and social support, which can influence infant-care and feeding practices(4,5). The Action Against Stunting Hub observational Study (AASH) (6) will follow a cohort of mother-infant dyads from pregnancy to 24-months post-partum in Hyderabad, India, East Lombok, Indonesia and Kaffrine, Senegal where biological and environmental exposures and the prevalence of child-stunting differ, ranging from 26% in Kaffrine-District (Senegal), 28% in Telegana-State, (India) to 44% in East Lombok, (Indonesia)(7–9).

This study will investigate the contribution of factors affecting child growth and development through assessment of:

1) Anthropometric status of pregnant women, their infants and the infants' biological father,

2) Biochemical and dietary nutritional status of women during pregnancy and infants from 6-months of age,

3) Fetal growth and development,

4) IYCF practices and morbidity of the infants,

5) Perinatal maternal stress, depression and social support, and

6) Associations of environmental, biological and sociological factors on infant growth

Confidential: For Review Only

METHODS AND ANALYSIS

Prospective pregnancy cohort studies will be conducted in Hyderabad, India, East Lombok, Indonesia and Kaffrine, Senegal. Women will be recruited in the second (Senegal and Indonesia) or third (India-delays due to COVID-19) trimester of pregnancy. Their infants will be followed until 24-months of age. The selection of methods for the nutrition study was guided by a conceptual framework for stunted linear infant growth (Figure 1). Summaries of measurement indicators and timings of data collection are presented in Tables 1 and 2. All data, except dietary intake in India and Senegal will be recorded using a centralised data collection application (CommCare®, Dimagi, Inc., Cambridge, MA, USA).

Data collection

Anthropometric Assessment

Anthropometric measurements of growth and body composition will be conducted on pregnant women in the second and/or third trimester of pregnancy, from the infants' biological father, and on infants within 72 hours after birth and at 3, 6, 9, 12, 18 and 24-months of age by trained enumerators (Table 2). Measurements will be done in duplicate, using standardised procedures and calibrated equipment, with a third measurement taken if the first and second measurements do not agree with the pre-defined limits (Table 3)(10).

Weight will be measured using the SECA-876 scale with a mother-child-function (range=0-250kg, precision±0.1kg). Adult height (standing and sitting) and infant length will be measured using a portable UNICEF-height/length board (range=0-221cm, precision±0.1cm), and for sitting height using a purpose-built anthropometric box(11). Mid-upper arm circumference (MUAC) and infant head circumference will be measured using the Lufkin-WP601 tape-measure. Both infant and adult arm measurements will be done on the left-hand side. Knee-to-heel length will be measured using a segmometer (Malby labs-India and Indonesia and Sealy-Senegal). Skinfolds measurements will be taken using a Holtain skinfold-caliper (range 0-40mm, precision±0.2mm).

In the data cleaning process consistency checks in CommCare for infant z scores; length-for-age (<-6 or >6sd), weight-for-length (<-5 or >5sd) and weight-for-length (<-6 or >5sd)(10,12), and for a negative difference between the current and preceding measurement of infant weight and length will be done. Z-scores will be calculated using the WHO child growth standards software(13). Prior to data collection, in each country, a standardisation session will take place to calculate intra- and

Confidential: For Review Only

inter-observer absolute and relative technical error of measurements and coefficients of reliability for all anthropometric measurements (9,11). Regular refresher training sessions will be conducted and, field supervisors will monitor data collection, using a checklist based on the study's Standard Operating Procedures (SOP).

Biochemical assessment of blood samples

Using standard procedures(14), blood samples will be collected from pregnant women (i.e., 10 mL in India and Senegal and 7 mL in Indonesia) during the third trimester (and second trimester in a sub-sample of women enrolled in a concurrent egg intervention trial in Indonesia). In Infants, 3 mL in India and Senegal and 2.5 mL in Indonesia of non-fasting blood samples will be collected at 6 months of age (and at 18-months in a sub-sample of children enrolled in an egg intervention study in India). The samples will be collected using Becton-Dickinson vacutainers®(EDTA and trace-element free). A complete blood count and HbA1C (women only) will be analysed on whole blood samples within 4-8 hours of collection. Blood samples will then be processed for separation of plasma, red blood cells and serum and aliquoted into microtubes for storage. All aliquots will be stored at -80ºC until analyses. The time of blood sample collection and separation will be recorded. The risks of contamination/analyte deterioration will be minimised by wearing powder-free gloves, keeping samples cool and protection from light.

Blood samples will be analysed for maternal and infant biochemical indicators of nutritional status, inflammation, and growth factors in infants (Table 4). Certified reference materials (CRM) will be included in the analyses where available to ensure analytical accuracy, precision and reliability. A certified pooled plasma sample (NIST-909c) will also be analysed across all laboratories to ensure inter-country comparability and analytical reliability.

Dietary intakes

Four-pass 24-hour dietary recall (MP24HR)(15) will be used to assess dietary intake. During the second (Indonesia and Senegal) and third trimesters (all sites), women will be asked to recall all foods and beverages consumed in a preceding 24 hours. The infants' dietary intakes from complementary foods will be estimated using and interactive 24-hour recall at 6, 9, 12, 18 and 24-months of age. For both mothers and children, a repeat recall will be collected on a randomly selected sub-sample of the population (i.e., approximately 10%) to estimate intra-subject variability in nutrient intakes. The nutrient density of the complementary foods will be compared to a desired nutrient density(16). For children ≥12 mo. of age, breast milk intakes will be estimated as the difference between mean estimated energy requirements and the mean energy intakes from

https://mc.manuscriptcentral.com/bmjpo

Confidential: For Review Only

complementary foods to estimate total daily intakes of energy and nutrients. The percentage of mothers and 1-year old children at risk of inadequate nutrient intakes will be estimated, depending on the nutrient, using the Estimated Average Requirement fixed cut-point approach or the full probability approach Quantities of foods and drinks consumed will be estimated by weighing actual foods/beverages or food models using calibrated digital electronic kitchen scales (Salter, 1024,-India and Senegal and Tanita, KD-160-Indonesia; precision±1.0 g), dimension measurements (cm), size estimations (small, medium or large) or using photographs, depending on the item. Details on sources of food acquisition will be recorded, and questions will be asked about whether their food intake on the preceding 24-hours was typical of their usual intakes, and for infants, whether they were breastfed.

Local conversion factors to convert portion size estimates to grams consumed and average recipe data to convert the quantity of a mixed-dish consumed into grams of individual ingredients will be developed when they are not available from previous studies.

In Indonesia, MP24HR data will be entered on a tablet (CommCare-application)(17), whilst in India and Senegal, a paper form will be used to collect dietary data from respondents and entered on a web-based application created specifically for this project and then uploaded on to the study data repository. Both applications (paper and tablet-format) will calculate each participant's intakes of energy and nutrients using the local conversion factors, average recipe data and country-specific food composition tables (FCT) in India and Indonesia(17,18), and a FCT developed using a hierarchy of other food composition values in Senegal(19–25). Paper-based forms will be reviewed immediately after data collection by the field supervisors and any questions resolved.

Ultrasound

Pregnant women in all three countries will have an antenatal ultrasound in government-recognised diagnostic centers at 32-weeks (± 2-weeks) to evaluate fetal growth and development(26). A trained obstetric sonographer will use a ultrasound system(27) to take the following measurements in triplicate, which will be checked by a radiologist; biparietal diameter, occipito-frontal diameter, head circumference, antero-posterior abdominal diameter, transverse abdominal diameter, abdominal circumference and femur length, fetal presentation, placental localisation and amniotic fluid volume, fetal movement, cardiac pulsation and the presence of congenital anomalies(28). Estimated gestational age will be calculated using the crown-rump length(29). The ultrasound report will be shared with the participant and advised to see the on-call doctor if necessary.

IYCF practices during the first 6 months after birth and breastmilk nutrient composition

Confidential: For Review Only

Within 72-hours of birth, enumerators will administer a questionnaire to mothers to evaluate: 1)pre-lacteal feeding (other feeds, besides breastmilk), 2)breastfeeding initiation and timing, 3)colostrum fed to the infant, 4)other feeds, besides breastmilk, and 5)infant nutrition supplements(30,31). Once a month until the infant is 6-months of age, the enumerator will ask mothers whether their infant has consumed anything other than breastmilk during the previous four-weeks. If the infant was not exclusively breastfed, detailed questions about the type of newly introduced liquids or solids will be asked.

A morning (between 6h00-12h00) breastmilk sample will be collected from mothers at 3-months post-partum by asking them to express all milk from one breast into a sterile collection bottle, using a manual breastmilk pump, following standard procedures(32). After collection the bottles will be placed in an ice-box and transported to a laboratory, where milk samples will be mixed with a vortex-mixer and aliquot 10 mL into three amber-coloured screw cap 15mL Eppendorf-tubes® (fat- and water-soluble vitamins and fatty acids analyses), one clear trace-element free tube (minerals analysis) and 1 mL in clear 1.5 mL Eppendorf-tubes (oligosaccharides analysis)(30). Samples will be immediately stored at -80°C (Table 2). CRM (NIST-1549a) – a standard control sample, will be included in the analyses to ensure analytical accuracy, precision and reliability.

Child morbidity

Child morbidity will be recorded using an adapted country-specific pictorial diary from birth to 24 months of age (33). Mothers will be asked to complete the diary daily in India and Indonesia and collected weekly. Enumerators will help complete any incomplete forms. In Senegal, morbidity data will be collected weekly via enumerator-led telephone interviews. The same information will be collected across all three countries. Mothers will be asked if her child was healthy or had active, reduced appetite, diarrhoea (with or without blood and/or mucus in the stools), vomiting, bloated stomach, fever, cough (with or without nasal discharge and/or wheezing), ear pain, chicken pox or measles(33).They will also record any health center visits and any medication the infant received. Additional morbidity questions (1-week recall) will also be asked at the same time as monthly IYCF questionnaire up to 6-months of age.

Maternal mental health and social support

Maternal stress will be assessed using cortisol level in hair samples (34) and the Global Measure of Perceived Stress Scale (GMPSS) questionnaire(35). Hair samples will be collected and GMPSS questionnaire administered during pregnancy (i.e., second trimester in Indonesia and Senegal and third trimester in India) and at 3 and 9 months post-partum. Hair grows approximately 1cm/month,

Confidential: For Review Only

therefore 3cm of hair will be collected as close as possible to the scalp from the posterior vertex of the head, placed on a hair collection card indicating the root end, wrapped in aluminum foil and stored in a dry, dark environment at room temperature until analysed, as described elsewhere(34,36). Information on hair-care practices, which influence the hair-cortisol measurements, will also be recorded(36). The GMPSS consists of 14 questions, on thoughts and feelings, which participants will rate using a 5-point Likert scale (35).

Maternal depression will be measured using the Edinburgh Postnatal Depression Scale (EPDS) and the Self-Reporting Questionnaire (SRQ) during the second (Senegal and Indonesia) and third trimester (India), and then in all countries at 3, 9 and 18-months post-partum(36). The EPDS consists of 10 statements, where participants are asked to rate how they felt in the last 7-days on a four-point Likert scale(37). As well as the classic EPDS, the new shorter version of the EPDS will also be asked to validate it against the classic EPDS in the three countries(39). The SRQ was developed by the WHO to screen for common mental disorders(40). It consists of 20 questions with dichotomous yes/no answers.

Maternal social support will be assessed using the Multidimensional Scale of Perceived Social Support(41) at 3, 9 and 18-months after birth. It includes 12 questions where participants are asked to rate how strongly they agree or disagree on a 7-point Likert scale(41), and their responses are recorded.

Statistical analysis plan

The statistical analysis of the Action Against Stunting Hub Study is explained in detail in the main protocol paper (6).

Patient and public partnership statement

Patients and public were not involved in the design of this study

Discussion

Addressing early life undernutrition is a public health priority in LMICs given its detrimental impacts on child survival, growth and development. The full extent and aetiology of undernutrition, however, are not well established. given the limited number of cohort studies from fetal life through to early childhood. As such studies are needed to understand the complex interplay among environmental and intrinsic physiological factors influencing early life nutritional status in different contexts.

Confidential: For Review Only

This study and others within the Action Against Stunting Hub (AASH) will investigate these complex relationships across three distinct geographies in sub-Saharan Africa and Asia. The analysis will examine factors affecting infant growth through three distinct periods; fetal period, 0-6 months of age and during the complementary feeding (6-24 months of age). The selection of its data collection protocols is underpinned by a theoretical framework of factors influencing infant and childhood growth (Figure 1), which will be further expanded through AASH inter-disciplinary collaborations to include influences of epigenetics, gut-health and food-systems. This study will also contribute to less-researched areas such as the influence of maternal depression, maternal stress and social support on infant care and feeding practices which ultimately affect infant-growth.

There are several limitations to this study. Firstly, although identical indicators of micronutrient status will be analysed across countries, the methods of analyses for two biochemical indicators differ. To minimize the influence of inter-laboratory assay differences on cross-country comparisons, a pooled-sample and CRMs, where available, will be analysed in all laboratories. Secondly, inter-population differences in maternal literacy necessitated a change in morbidity data collection protocols in Senegal. Thirdly, when investigating causal inferences, there are limitations with a prospective cohort study design, including loss-to-follow-up bias and confounding. These biases will be minimised through concerted sensitisation/motivational efforts to reduce loss-to-follow-up and the use of Directed-Acylic-Graphs.

Conclusions

Results from the nutrition study conducted in three diverse geographic regions will form part of the 'whole child approach' pursued under AASH. The interdisciplinary collaborations established across the hub provide a unique opportunity to investigate the complex inter-relationships among environmental and intrinsic physiological factors influencing early life growth and development. Such insights are important for informing effective intervention design and the policy actions needed to support global efforts in reducing early child-stunting.

Confidential: For Review Only

Figure 1: A conceptual framework of pathways to assess the impact of nutrition, nutritional status and maternal wellbeing on child stunting

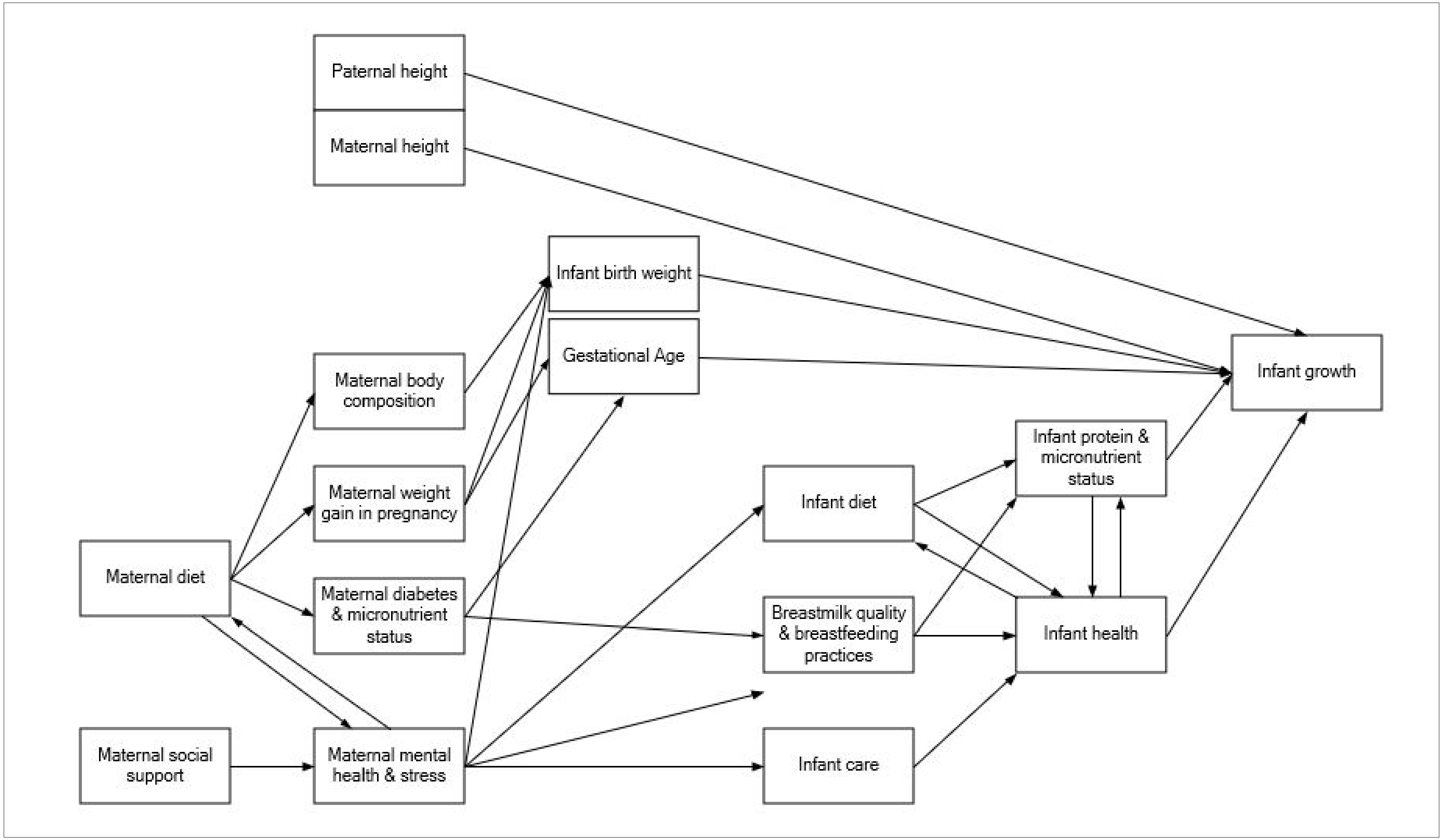

Table 1: Flow chart for data collection timing

Measurements	2nd trim[1]	3rd trim	Birth	1-2 mo[2]	3 mo	4-5 mo[2]	6 mo	9 mo	12 mo	18 mo	24 mo
**Anthropometry**											
Pregnant women	IS[3]	All[4]									
Women weight only				All	All	All	All	All	All	All	All
Biological fathers			All								
Infants			All		All		All	All	All	All	All
Infants weight only				All		All					
**Blood Sample**											
Pregnant women	IS	I[5]									
Infants							All				
**Dietary Intakes**											
Pregnant women	IS	All									
Infants							All	All	All	All	All
**Ultrasound**		All									
Pregnant women											
**Breastfeeding**											
Breast milk sample					All						
Questionnaires			All	All	All	All	All				
**Morbidity[6]**			All	All	All	All	All	All	All	All	All
**Mental Health**											
Hair cortisol	IS	I			All						
GMPSS[7]	IS	I			All			All		All	
EPDS[8]	IS	I			All			All		All	
MSPSS[9]	IS	I			All			All		All	
SRQ[10]		All			All			All			

[1]–trimester; [2] – data collected each month; [3] – Indonesia and Senegal only; [4] – all three countries; [5] - India only; [6] – infant morbidity data is recorded everyday; [7] - Global Measure of Perceived Stress Scale (GMPSS); [8] - Edinburgh Postnatal Depression Scale (EPDS); [9] - Multidimensional Scale of Perceived Social Support (MSPSS), [10] Self-Reporting Questionnaire (SRQ)

Confidential: For Review Only

Table 2: Summary of the data that will be collected from each participant and the main indicators generated for statistical analysis.

Data collection	Main Indicators
**Anthropometry**	
Pregnant women: Weight, height, sitting height, mid-upper-arm-circumference (MUAC) and skinfolds (triceps, biceps, sub-scapular)	***Pregnant women***: Weight gain in pregnancy [1]**(con)**, sitting height to standing height ratio**(con)** and arm muscle and fat areas **(con)**
Women post-partum: Weight	
Biological father: Weight, height, sitting height, MUAC and skinfolds (triceps, biceps, sub-scapular)	***Maternal:*** Post-partum body-mass index (BMI) [2]**(cat)** and % under-weight/over-weight /obese **(con)**
Infant: Birth weight, birth length, head circumference, knee-to-heel length, weight, length, MUAC, skinfolds (triceps, sub-scapular)	***Paternal:*** BMI **(cat)**, % under-weight/over-weight /obese **(con)**, sitting height to standing height ratio **(con)**, and arm muscle and arm fat areas **(con)**<br>***Infant***: Birth weight **(con)**, % low birth weight **(con)**, Z-scores[3] for weight-for-age **(cat)**, weight-for-length **(cat)**, length-for-age **(primary outcome-cat)**, length **(primary outcome-con)**, BMI **(cat)**, skinfolds **(con)**, arm and head circumferences **(con)**, Z-scores[3] for velocity-for-length **(primary outcome-con)**, weight and head circumference **(con)**, Z-score for weight-for-length[3] **(con), %** stunted **(con)**, % wasted **(con)** and % low arm circumference **(con).**
**Blood Sample**	
Pregnant women: Blood sample in (1) BD EDTA lavender tube (6 ml India and Senegal and 5.75 ml Indonesia); (2) BD royal blue top trace element free tube (4 ml India, 1 ml Indonesia and 5 ml Senegal); and (3) BD microtainer (120 µl India and Senegal and 250 µl Indonesia)	***Pregnant women***: (1) Micronutrient status (RBC folate, Plasma folate**,** B12, homocysteine, methylmalonic acid, choline, betaine, complete blood count, ferritin, transferrin receptor, hepcidin, retinol binding protein, retinol, amino acids, Fe, Zn, Cu, Se, Ca, Cr, Cd, Co, Pb, Hg, As, Al, and 25-dihydroxyvitamin D) **(con)**; (2) RBC-fatty acids **(con)**; (3) Gestational Diabetes with HbA1C **(cat)**; and (4) Inflammation with C-reactive protein, α1-acid glycoprotein **(con)**
Infants: Non-fasting blood sample in (1) BD EDTA lavender tube (2.5 ml India and Senegal and 1.5 ml Indonesia); (2) BD royal blue top	***Infants***: Micronutrient status (Folate, B_{12}, methylmalonic acid, choline, betaine, complete blood count, ferritin, transferrin receptor, hepcidin, retinol binding protein, retinol, RBC-fatty

Confidential: For Review Only

trace element free tube (0.7 ml India, 1 ml Indonesia and 2.5ml Senegal); and (3) BD microtainer (20 µl India, and 40 µl Senegal)	acids and Folate, Fe, Zn, Cu, Se, Ca, Cr, Cd, Co, Pb, Hg, As, Al) **(con);** (2) RBC-fatty acids **(con)**; (3) Amino acids **(con)**; (4) Growth Hormone, Insulin-like growth factor-1, IGF binding protein, intestinal fatty acid binding protein **(con)**, and (5) Inflammation with C-reactive protein, α1-acid glycoprotein **(con)**
**Dietary Intakes**<br>***Pregnant women:*** Multiple pass 24-hour diet recall with repeat in 10% of women at each time point<br>***Infants:*** Multiple pass 24-hour diet recall with repeat in 10% of infants at each time point	 ***Pregnant women:*** Intakes of selected foods/beverages (g/d) **(con)**, energy and nutrients **(con)**, % at risk of inadequate nutrient intakes **(con)**, % energy from selected food groups and food sub-groups **(con)**<br>***Infants:*** Intakes of selected foods/beverages (g/d), energy and nutrients **(con)**, the nutrient density of complementary feeding diets in relation to desired levels **(con)**, % energy from selected food groups and food sub-groups **(con)**, % continued breastfed to 24-months of age **(con),** complementary feeding (introduction of solid, semi-solid or soft foods 6–8 months, minimum dietary diversity 6–23 months, minimum meal frequency 6–23 months, minimum milk feeding frequency for non-breastfed children 6–23 months, minimum acceptable diet 6–23 months, egg and/or flesh food consumption 6–23 months, sweet beverage consumption 6–23 months, unhealthy food consumption 6–23 months and zero vegetable or fruit consumption 6–23 months)<br>***Infants ≥12-months:*** % at risk of inadequate nutrient intakes and nutrient density **(con)**
**Ultrasound**<br>**Pregnant woman and developing infant:** 1) Biparietal diameter, 2) occipito-frontal diameter, 3) head circumference, 4) antero-posterior abdominal diameter, 5) transverse abdominal diameter, 6) abdominal circumference (AC) and 7) femur length, 8) fetal presentation, 9) placental localization, 10) amniotic fluid volume,	 **Pregnant woman and developing infant:** Intrauterine growth retardation (IUGR), gestational age **(con)** and fetal-weight-for-gestational age **(con)**

Confidential: For Review Only

11) fetal movement, 12) cardiac pulsation, 13) presence of congenital anomalies and 14) gestational age (GA)	
**Breastfeeding**<br>***Breast milk sample:*** Full expression of a morning breastmilk sample from one breast<br><br>***Questionnaire at birth***: Administered within 72 hours of birth on initial breastfeeding practices<br>***Questionnaire in first 6-months***: Administered once per month on breastfeeding practices	***Breastmilk sample:*** the breastmilk content of fatty acids, fat- and water-soluble vitamins, minerals and oligosaccharides **(con)**<br><br>***Questionnaire at birth***: % ever breastfed **(con)**, % early initiation of breastfeeding **(con)**, % pre-lacteal feeding **(con)**<br><br>***Questionnaire in first 6-months***: % exclusively breastfed **(con)**, % mixed milk feeding **(con)**, age of introduction of semi-solid or solid foods **(con)**
**Infant Morbidity**<br>***Paper based pictorial chart:*** Completed daily by caregivers on infant health status, illness, clinic visits and medication use	***Paper based pictorial chart:*** % frequency and number of days of reduced appetite, stomach pain[4], bloated stomach[4], diarrhoea, vomiting fever, cough, wheezing, nasal discharge, ear pain[5], chicken pox[5] or measles[5], health center visits and prescribed medication **(con)**
**Maternal mental health& well-being**<br>***Hair samples:*** Collected from the posterior vertex of the head from pregnant women ***Questionnaires***: Administered to the woman during pregnancy and post-partum	Hair cortisol content as an indicator of stress (>182 pg/mg) **(con)**<br><br>Global Measure of Perceived Stress Scale (GMPSS) (0-13=low perceived stress, 14-26=moderate perceived stress and 27-40=high perceived stress) **(cat)**<br>Edinburgh Postnatal Depression Scale (EPDS) cut-off of 13 **(cat)**<br>Multidimensional Scale of Perceived Social Support (MSPSS) (12-35 indicates low perceived support, 36-60 indicates medium perceived support and 61-84 indicates high perceived support) **(cat)**

Confidential: For Review Only

[1]continuous **(con)**; [2]categorical **(cat)**

[3]Z-scores will be calculated utilizing the WHO child growth standards software (13), [4]Senegal only, [5]India and Indonesia only,

Confidential: For Review Only

Table 3: Pre-defined limits in the difference between first and second anthropometry measurements

Measure	Pre-defined limits
Adult and infant weight	0.1 kg
Adult height	0.5 cm
Adult Mid Upper Arm Circumference	0.5 cm
Adult skinfolds	0.2 cm/2 mm
Infant length	0.7 cm
Infant Head circumference	0.5 cm
Infant knee to heel	0.5 cm
Infant Mid Upper Arm Circumference	0.5 cm
Infant skinfolds	0.2 cm/2 mm

Confidential: For Review Only

Table 4: Analysis methods for parameters in India, Indonesia and Senegal

Parameters	India	Indonesia	Senegal
**Blood samples**			
Complete Blood Count<br>Glycated Haemaglobin (HbA1C -pregnant women only)	CBC auto-analyser (Horiba ABX Micros ES60)<br>HbA1C analyser (Affinion ™ Hb1Ac, Abbot)	CBC auto-analyser (SYSMEX XP100)<br>HbA1C analyser (PocketChem A1c, Arkray Inc)	CBC auto-analyser<br>HbA1C analyser (HPLC D10 Biorad)
Red Blood Cell folate<br>Vitamin D<br>Vitamin B12<br>Vitamin B9<br>Methylmalonic acid (MMA)<br>Choline<br>Betaine	LC-MS/MS[1]	LC-MS/MS	LC-MS/MS
Homocysteine	LC-MS/MS	HPLC[2]	HPLC
Red Blood Cell-Fatty acids	GC-FID[3]	GC-FID	GC-FID
Amino Acids	HPLC	LC-MS/MS	LC-MS/MS
Retinol	HPLC	HPLC	HPLC
Ferritin<br>TfR<br>C-reactive protein<br>α1-Acid glycoprotein<br>Hepcidin<br>Retinol binding protein<br>Insulin like growth factor-1<br>Insulin-like growth factor binding protein<br>Intestinal fatty-acid binding protein	ELISA[4]	ELISA	ELISA
Minerals/trace elements	ICP-MS[5]	ICP-MS	ICP-MS
Remaining Red blood cells<br>Remaining plasma	Kept at -80ºC	Kept at -80ºC	Kept at -80ºC
**Breastmilk samples**			
Fat-soluble vitamins A and D	HPLC/LC-MS[6]	HPLC/LC-MS	HPLC/LC-MS
Water-soluble vitamins B1, B2, B3, B6, B9 and B12	LC-MS[7]	LC-MS	LC-MS
Fatty acids	GC-FID	GC-FID	GC-FID
Minerals	ICP-MS	ICP-MS	ICP-MS
Oligosaccharides	SPEC[8] and HPLC	SPEC and HPLC	SPEC and HPLC
Remaining breastmilk	Kept at -80ºC	Kept at -80ºC	Kept at -80ºC

https://mc.manuscriptcentral.com/bmjpo

Confidential: For Review Only

Hair samples			
Cortisol	ELISA	ELISA	ELISA

[1] Liquid Chromatography with tandem Mass Spectrometry (LC-MS/MS), [2] High Performance Liquid Chromatography, [3] Gas Chromatography-Flame-ionisation detection (GC-FID),[4] Enzyme-Linked Immunoassay, [5] Inductively coupled plasma mass spectrometry, [6] High Performance Liquid Chromatography/ Liquid Chromatography-Mass Spectrometry [7] Liquid Chromatography-Mass Spectrometry, [8] Solid Phase Extraction Chromatography

Confidential: For Review Only

References

1. Lee RD, Nieman DC. Nutritional Assessment. International Edition. In: Forth Edition. New York: McGraw-Hill; 2007.

2. Zoleko-Manego R, Mischlinger J, Dejon-Agobé JC, Basra A, Mackanga JR, Diop DA, et al. Birth weight, growth, nutritional status and mortality of infants from Lambaréné and Fougamou in Gabon in their first year of lifeRella Zoleko-Manego,Johannes ,Jean Claude ,Arti Basra,J. Rodolphe Mackanga,Daisy Akerey Diop,Ayola Akim Adegnika,Selidji T. Agnandji,Bertrand Lell,Peter G. Kremsner,Pierre Blaise Matsiegui,Raquel González,Clara Menendez,Michael Ramharter,Ghyslain Mombo-Ngoma Published: February 9, 2021. PLOS ONE. 2021;16(2).

3. Woldeamanuel GG, Geta TG, Mohammed TP, Shuba MB, Bafa TA. Effect of nutritional status of pregnant women on birth weight of newborns at Butajira Referral Hospital, Butajira, Ethiopia. SAGE Open Med. 2019;7.

4. Black R, Allen L, Bhutta Z, Caulfield LE, de Onis M, Ezzati M. Maternal and child undernutrition: global and regional exposures and health consequences. Lancet. 2008;371(9608):243–60.

5. UNICEF. Nutrition, for every child UNICEF Nutrition Strategy 2020-2030. UNICEF Nutrition Section, Programme Division; 2020.

6. Heffernan C, et al. Developing a whole child approach for understanding stunting in early childhood: Introducing the UKRI GCRF Action Against Stunting Hub. Submitt Rev Process. 2022;

7. National Agency for Statistics and Demography - ANSD / Senegal and ICF. Demographic and Continuous Health Survey (EDS-Continue) 2017 [Internet]. Dakar, Senegal: ANSD and ICF; 2018. Available from: http://dhsprogram.com/pubs/pdf/FR345/FR345.pdf.

8. International Institute for Population Sciences. National Family Health Survey NFHS-4, 2015-16. India, Mumbai:IIPS; 2017.

9. Agency of Health Research and Development. Indonesia Basic Health Research 2018 . Indonesia; 2018.

10. de Onis M, et al. Measurement and standardization protocols for anthropometry used in the construction of a new international growth reference. Food Nutr Bullitin. 2004;25(S1):S27–36.

11. Norton K. International Standards for Anthropometric Assessment. International Society for the Advancement of Kinanthropometry; 2001.

12. WHO. RECOMMENDATIONS FOR DATA COLLECTION, ANALYSIS AND REPORTING ON ANTHROPOMETRIC INDICATORS IN CHILDREN UNDER 5 YEARS OLD. Geneva: World Health Organization and the United Nations Chhildren's Fund (UNICEF); 2019. Report No.: Licence: CC BY-NC-SA 3.0 IGO.

13. WHO: Geneva. WHO Anthro for personal computers, version 3.2.2, 2011: Software for assessing growth and development of the world's children. [Internet]. 2010. Available from: (http://www.who.int/childgrowth/software/en/

14. Centre for Disease Control and Prevention. National Health and Nutrition Examination Survey (NHANES) MEC Laboratory Procedures Manual. 2020.

15. Gibson RS, Ferguson EL. An Interactive 24-Hour Recall for Assessing the Adequacy of Iron and Zinc Intakes in Developing Countrie. Washington, DC, USA: International Life Sciences Institute; 2008.

16. Dewey KG, Brown K. Update on technical issues concerning complementary feeding of young children in developing countries and implications for intervention programs Kathryn G Dewey 1, Kenneth H Brown. Food Nutr Bull. 2003;24(2):239.

Confidential: For Review Only

17. Htet MK, Fahmida U, Do TT, Dibley MJ, Ferguson E. The used of tablet-based multiple-pass 24-hour dietary recall application (MP24Diet) to collect intake of children under two years old in the prospective cohort study in Indonesia. Nutrients. 2019;11(12):2889.

18. Data Komposisi Pangan Indonesia (Indonesian Food Composition Table). https://www.panganku.org/id-ID/beranda. 2018.

19. Longvah T, Anathan R, Bhaskarachary K, Venkalah K. Indian Food Composition Tables. National Institute of Nutrition, Indian Council of Medical Research, Department of Health Research, Hyderabad, India; 2017.

20. FAO/INFOODS. Food Composition Table for Western Africa (2019) /Table de composition des aliments FAO/INFOODS pour l'Afrique de l'Ouest. 2019.

21. Nigeria Food Database [Internet]. Available from: http://nigeriafooddata.ui.edu.ng/

22. Prynne C, Paul A. Food Composition Table for Use in The Gambia. Cambridge: Medial Research Council Human Nutrition Research; 2011.

23. SAFOODS, SA MRC. South African Food Data System [Internet]. 2018. Available from: http://safoods.mrc.ac.za/

24. Public Health England. McCance and Widdowson's The Composition of Foods Integrated Dataset 2021. 2021. Report No.: GW-2010.

25. US Department of Agriculture. Food and Nutrition Database [Internet]. Available from: https://fdc.nal.usda.gov/

26. Salomon L, Alfirevic Z, Da Silva-Costa F. ISUOG Practice Guidelines: ultrasound assessment of fetal biometry and growth. Ultrasound Obstet Gynecol. 2015;46(4):391–7.

27. Phillips Affiniti 50 Ultrasound system [Internet]. Available from: https://www.philips.co.in/healthcare/product/HC795208/affiniti-50-ultrasound-system

28. Romosan G, Henriksson E, Rylander A, Valentin L. Diagnostic performance of routine ultrasound screening for fetal abnormalities in an unselected Swedish population in 2000–2005. Ultrasound Obstet Gynecol. 2009;34(5):526–33.

29. Gadsbøll, K, Wright A, Kristensen SE, Verfaille V, Nicolaides K, Wright D, et al. Crown–rump length measurement errors: impact on assessment of growth. Ultrasound Obstet Gynecol. 2021;58(3):354–9.

30. Caulfield LE, Bose A, Chandyo RK, Nesamvuni C, de Moraes ML, Turab A, et al. Infant Feeding Practices, Dietary Adequacy, and Micronutrient Status Measures in the MAL-ED Study. Clin Infect Dis. 2014;59 Suppl 4:S248-54.

31. WHO. Indicators for assessing infant and young child feeding practices. Part 2 Measurement. Geneva, Switzerland; 2010.

32. Leghi GE, Middleton PF, Netting MJ, Wlodek ME, Geddes DT, Muhlhausler BS. A Systematic Review of Collection and Analysis of Human Milk for Macronutrient Composition. Methodol Math Model. 2020;00:1–19.

33. Richard SA, Barrett L, Guerrant R. Disease surveillance methods used in the 8-stie MAL-ED cohort study. Clin Infect Dis. 2014;59(Suppl 4):S220–4.

34. Greef M, Levine J, Abuzgaia A, Elzagallaai A. Hair cortisol analysis: An update on methodological considerations and clinical applications. Clin Biochem. 2019;63:1–9.

35. Cohen S, Kamarck T, Mermelstein R. A global measure of perceived stress. J Health Soc Behav. 1983;24:385–96.

Confidential: For Review Only

36. Wright K, Ford J, Perazzo J, Jones L, Mahan S, Sullenbarger B, et al. Collecting Hair Samples for Hair Cortisol Analysis in African Americans. J Vis Exp. 2018;136(e57288):1–7.

37. Cox JL, Holden JM, Segovsky R. Edinburgh Postnatal Depression Scale (EPDS). Br J Psychiatry. 1987;150.

38. Harding T, DeArango V, Baltazar J. Mental disorders in primary health care: a study of their frequency and diagnosis in four developing countries. Psychol Med. 1980;10:231–41.

39. van Heyningen T, Myer L, Tomlinson M, Honikman S. The development of an ultra-short, maternal mental health screening tool in South Africa. Glob Ment Health. 2019;6(e24):1–14.

40. Beusenberg M, Orley JH. A User's guide to the self reporting questionnaire: A User's guide to the self reporting questionnaire [Internet]. Geneva: World Health Organisation. Division of Mental Health; 1984. Available from: https://apps.who.int/iris/handle/10665/61113

41. Zimet GD, Dahlem NW, Zimet SG, Farley GK. The Multidimensional Scale of Perceived Social Support. J Pers Assess. 1988;52:30–41.

Confidential: For Review Only

Authors' contributions: HDK and ELF were responsible for the overall design, training and overseeing implementation of the research. UF, MKH, BK, BF, RM, RP, RP, SF, SD, RP, SA, AW, JW, PH, CH were involved in its design. UF, MKH, BK, BF, DY, DS, NLZ, TCA, RM, RP, RP, SFR, KS, DP, SK, DY, SD, PL-S, BD, PM, SF, ID, AD, TVI, FT, AD, SS, BMK, DTT implemented the research. HD-K and ELF wrote the manuscript. All authors read, provided comments on and approved the final version of the manuscript.

Funding: This study is funded by UKRI Global Challenges Research Fund (GCRF) (No. MR/S01313X/1). The views expressed in this work are those of the authors and not the funder.

Competing interest statement: The authors declare no conflict of interest.

Confidential: For Review Only

BMJ Paediatrics Open

Anthropometric, biochemical, dietary, morbidity and wellbeing assessments in women and children in Indonesia, India and Senegal: a UKRI GCRF Action Against Stunting Hub protocol paper

Journal:	*BMJ Paediatrics Open*
Manuscript ID	bmjpo-2022-001683.R2
Article Type:	Protocol
Date Submitted by the Author:	22-Mar-2023
Complete List of Authors:	Davies-Kershaw, Hilary; LSHTM, Department of Population Health<br>Fahmida, Umi; SEAMEO RECFON<br>Htet, Min Kyaw; SEAMEO RECFON<br>Kulkarni, Bharati; National Institute of Nutrition<br>Faye, Babacar; UCAD, Service de Parasitologie-Mycologie- Pédiatrie, Faculté de médecine<br>Yanti, Dwi; SEAMEO RECFON<br>Shinta, Dewi; SEAMEO RECFON<br>Zahra, Nur ; SEAMEO RECFON<br>Angelin, Tiffany C.; SEAMEO RECFON<br>Madhari, Radhika; National Institute of Nutrition<br>Pullakhandam, Raghu; National Institute of Nutrition<br>Palika, Ravindranadh; National Institute of Nutrition<br>Dasi, Teena; National Institute of Nutrition<br>Fernandez Rao, Sylvia; National Institute of Nutrition<br>B, Santosh Kumar; National Institute of Nutrition<br>Selvaraj, Kiruthika; National Institute of Nutrition<br>Palepu, Dharani; National Institute of Nutrition<br>Yadev, Dinesh; LIDC<br>Diouf, Saliou; UCAD, Service de Parasitologie-Mycologie- Pédiatrie, Faculté de médecine<br>Lopez-Sall, Philomene; UCAD, Service de Parasitologie-Mycologie-Pédiatrie, Faculté de médecine<br>Diallo, Babacar; UCAD, Service de Parasitologie-Mycologie- Pédiatrie, Faculté de médecine<br>Mouissi, Princillia; UCAD, Service de Parasitologie-Mycologie- Pédiatrie, Faculté de médecine<br>Fall, Sally; UCAD, Service de Parasitologie-Mycologie- Pédiatrie, Faculté de médecine<br>Diallo, Ibrahima; UCAD, Service de Parasitologie-Mycologie- Pédiatrie, Faculté de médecine<br>Djigal, Aicha; UCAD, Service de Parasitologie-Mycologie- Pédiatrie, Faculté de médecine<br>Immerzeel, Tabitha; LSHTM, Department of Population Health<br>Tairou, Fassia; UCAD, Service de Parasitologie-Mycologie- Pédiatrie, Faculté de médecine

	Diop, Assana; UCAD, Service de Parasitologie-Mycologie- Pédiatrie, Faculté de médecine<br>Pradeilles, Rebecca; Loughborough University, School of Sport, Exercise and Health Sciences; MOISA, UMR<br>Strout, Sara; LSHTM, Department of Population Health<br>Momo Kadia, Benjamin; Liverpool School of Tropical Medicine Department of Clinical Sciences,<br>Tata, Darius; LIDC<br>Jobarteh, Modou; London School of Hygiene & Tropical Medicine, Epidemiology & Population Health<br>Allen, Stephen; LSTM, Department of Clinical Sciences<br>Walker, Alan; University of Aberdeen,<br>Webster, Joanne P.; University of London, Department of Pathobiology and Population Sciences<br>Haggarty, Paul; University of Aberdeen<br>Heffernan, Claire; LIDC<br>Ferguson, Elaine; LSHTM, Department of Population Health
Keywords:	Epidemiology, Growth, Data Collection

SCHOLARONE™
Manuscripts

I, the Submitting Author has the right to grant and does grant on behalf of all authors of the Work (as defined in the below author licence), an exclusive licence and/or a non-exclusive licence for contributions from authors who are: i) UK Crown employees; ii) where BMJ has agreed a CC-BY licence shall apply, and/or iii) in accordance with the terms applicable for US Federal Government officers or employees acting as part of their official duties; on a worldwide, perpetual, irrevocable, royalty-free basis to BMJ Publishing Group Ltd ("BMJ") its licensees and where the relevant Journal is co-owned by BMJ to the co-owners of the Journal, to publish the Work in this journal and any other BMJ products and to exploit all rights, as set out in our licence.

The Submitting Author accepts and understands that any supply made under these terms is made by BMJ to the Submitting Author unless you are acting as an employee on behalf of your employer or a postgraduate student of an affiliated institution which is paying any applicable article publishing charge ("APC") for Open Access articles. Where the Submitting Author wishes to make the Work available on an Open Access basis (and intends to pay the relevant APC), the terms of reuse of such Open Access shall be governed by a Creative Commons licence – details of these licences and which Creative Commons licence will apply to this Work are set out in our licence referred to above.

Other than as permitted in any relevant BMJ Author's Self Archiving Policies, I confirm this Work has not been accepted for publication elsewhere, is not being considered for publication elsewhere and does not duplicate material already published. I confirm all authors consent to publication of this Work and authorise the granting of this licence.

Confidential: For Review Only

Anthropometric, biochemical, dietary, morbidity and wellbeing assessments in women and children in Indonesia, India and Senegal: a UKRI GCRF Action Against Stunting Hub protocol paper

Authors

Hilary Davies-Kershaw[1], Umi Fahmida[2], Min Kyaw Htet[2], Bharati Kulkarni[3], Babacar Faye[4],Dwi Yanti[2], Dewi Shinta[2], Nur Lailatuz Zahra[2], Tiffany Cornelia Angelin[2], Radhika Madhari[3], Raghu Pullakhandam[3], Ravindranadh Palika[3], Teena Rajeev[3] ,Sylvia Fernandez Rao[3], Santosh Kumar Banjara[3] ,Kiruthika Selvaraj[3], Dharani Pratyusha[3], Dinesh Yadav[3,9],Saliou Diouf[4], Philomène Lopez-Sall[4], Babacar Diallo[4], Princillia Mouissi[4], Sally Fall[4], Ibrahima Diallo[4], Aicha Djigal[4], Tabitha Van Immerzeel[1], Fassia Tairou[4], Assana Diop[4], Rebecca Pradeilles[5], Sara Strout[1], Benjamin Momo Kadia[6], Darius Tetsa, Tata[9], Modou Lamin Jobarteh[9], Stephen Allen[6], Alan Walker[7], Joanne Webster[8], Paul Haggarty[7], Claire Heffernan[9], Elaine Ferguson[1]

Authors' affiliations

[1]Department of Population Health, Faculty of Epidemiology and Population Health, London School of Hygiene and Tropical Medicine (LSHTM) United Kingdom (UK)

[2]Southeast Asian Ministers of Education Organization Regional Center for Food and Nutrition (SEAMEO RECFON), Jakarta, Indonesia

[3]ICMR - National Institute of Nutrition, Hyderabad, India

[4]Service de Parasitologie-Mycologie- Pédiatrie, Faculté de médecine, Université Cheikh Anta Diop (UCAD), Dakar, Senegal.

[5]UMR MoISA, University of Montpellier, France

[6]Department of Clinical Sciences, Liverpool School of Tropical Medicine (LSTM), Liverpool, UK

[7]Rowett Institute, University of Aberdeen, Aberdeen, UK

[8]Department of Pathobiology and Population Sciences, Royal Veterinary College, University of London, Hawkshead Campus, Herts, UK

[9]London International Development Centre (LIDC), London, UK

Corresponding author

Hilary Davies-Kershaw, Department of Population Health, LSHTM, email: hilary.davies@lshtm.ac.uk

Confidential: For Review Only

ABSTRACT

Introduction: Child-stunting has a complex aetiology, especially in the first 1000 days of life. Nutrition interventions alone have not produced expected impacts in reducing/preventing child-stunting, indicating the importance of understanding the complex interplay between environmental, physiological and psychological factors influencing child nutritional status.

This study will investigate maternal and child nutrition, health and wellbeing status and associated factors through the assessment of:(1) anthropometry; (2) biomarkers of nutrition and health status; (3) dietary intakes; (4) fetal growth and development; (5) infant morbidity; (6) Infant and Young Child Feeding (IYCF); and (7) perinatal maternal stress, depression and social support.

Methods:

This study will be conducted in a prospective pregnancy cohort in India, Indonesia and Senegal. Pregnant women will be recruited in the second (Indonesia, Senegal) and third (India) trimester of pregnancy, and the mother and infant dyads followed until the infant is 24-months of age. During pregnancy, anthropometric measures will be taken, venous blood samples will be collected for biochemical assessment of nutrition and health status, dietary intakes will be assessed using a 4-pass-24-hour dietary recall method (MP24HR), fetal ultrasound for assessment of fetal growth. After birth, anthropometry measurements will be taken, venous blood samples will be collected, MP24HR will be conducted, infant morbidity and IYCF practices will be assessed and a sample of breastmilk will be collected for nutrient composition analyses. Perinatal maternal stress, depression, social support and hair cortisol levels (stress) will be measured. The results from this study will be integrated in an interdisciplinary analysis to examine factors influencing infant growth and inform global efforts in reducing child stunting.

Ethics and dissemination*:*

Ethical approval was granted by the Ethics Committee of the London School of Hygiene and Tropical Medicine (17915/RR/17513); National Institute of Nutrition (ICMR)- Ministry of Health and Family Welfare, Government of India (CR/04/I/2021); Health Research Ethics Committee, University of Indonesia and Cipto Mangunkusumo Hospital (KET-887/UN2.F1/ETIK/PPM.00.02/2019); and the Comité National d'Ethique pour la Recherche en Santé, Senegal (Protocole SEN19/78); the Royal Veterinary College (URN SR2020-0197); and the International Livestock Research Institute Institutional Research Ethics Committee (ILRI-IREC2020-33). Results will be published in peer-reviewed journals and disseminated to policy makers and participating communities.

Confidential: For Review Only

Keywords:

Stunting, nutritional status, anthropometry, dietary intake, micronutrient status, breastmilk, infant morbidity, maternal mental health and social support

SUMMARY BOX

What is already known on this topic?

- The global burden of child stunting remains unacceptably high
- Whilst some progress has been made, reduction of the burden of child stunting is very slow and sometimes unresponsive
- Increasing the understanding of the complex aetiology of child stunting is critical to programmes and policies to further prevent or reduce the global burden

What this study hopes to add?

- Discern the contribution of maternal and child nutrition and nutritional status on child stunting as part of an interdisciplinary analysis
- Increase understanding of complex interrelationships and biological factors that influence maternal and child nutrition and stunting
- Contribute to an understanding of the influence of maternal depression, maternal stress and social support on child nutritional status

How this study might affect research, practice or policy

- Inform policy making by determining and comparing risks of undernutrition across the three settings using anthropometric, biochemical and dietary nutritional status for pregnant women and their offspring

Confidential: For Review Only

INTRODUCTION

Adequate nutrition during the first 1000 days of life (from conception to 24-months of age) is essential for optimal linear and ponderal growth, immune function and cognitive-development.[1, 2] Poor maternal nutritional status during pregnancy and lactation contributes to infant undernutrition through its influence on infant birthweight and length, gestation length, infant immunity and the nutrient content of breastmilk.[1, 3] Important intermediate factors contributing to undernutrition after birth include the quality of infant-care and breastfeeding practices, infant's dietary intake, and the frequency and severity of infant illnesses.[4] Along with the widely recognised underlying causes of undernutrition, including food-security, poverty, maternal education and health service infrastructure,[4] there is a growing interest in less-researched areas such as maternal depression, stress and social support, which can influence infant-care and feeding practices.[4, 5] The Action Against Stunting Hub observational Study (AASH) will follow a cohort of mother-infant dyads from pregnancy to 24-months post-partum in Hyderabad, India, East Lombok, Indonesia and Kaffrine, Senegal where biological and environmental exposures and the prevalence of child-stunting differ, ranging from 26% in Kaffrine-District (Senegal), 28% in Telegana-State, (India) to 44% in East Lombok, (Indonesia).[6–8]

This study will investigate the contribution of factors affecting child growth and development through assessment of:

1) Anthropometric status of pregnant women, their infants and the infants' biological father,

2) Biochemical and dietary nutritional status of women during pregnancy and infants from 6-months of age,

3) Fetal growth and development,

4) Infant and Young Child Feeding (IYCF) practices and morbidity of the infants,

5) Perinatal maternal stress, depression and social support, and

6) Associations of environmental, biological and sociological factors on infant growth

Confidential: For Review Only

METHODS AND ANALYSIS

Prospective pregnancy cohort studies will be conducted in Hyderabad, India (n=717), East Lombok, Indonesia (n=702) and Kaffrine, Senegal (n=763). The sample size and power were calculated to test 100 independent epigenetic signatures between stunted and non-stunted children (Epigenetic Studies in Children at Risk of Stunting and their Parents in India, Indonesia, and Senegal: a UKRI GCRF Action Against Stunting Hub protocol paper BMJ Paeds Open Ramsteijn et al, in press). Women will be recruited in the second (Senegal and Indonesia) or third (India-delays due to COVID-19) trimester of pregnancy. Their infants will be followed until 24-months of age. The selection of methods for the nutrition study was guided by a conceptual framework for stunted linear infant growth (Figure 1). Summaries of timings of data collection and measurement indicators and timings of data collection are presented in Supplementary Tables 1 and 2 respectively. All data, except dietary intake in India and Senegal will be recorded using a centralised data collection application (CommCare®, Dimagi, Inc., Cambridge, MA, USA).

Data collection

Anthropometric Assessment

Anthropometric measurements of growth and body composition will be conducted on pregnant women in the second and/or third trimester of pregnancy, from the infants' biological father, and on infants within 72 hours after birth and at 3, 6, 9, 12, 18 and 24-months of age by trained enumerators (Supplementary Table 2). Measurements will be done in duplicate, using standardised procedures and calibrated equipment, with a third measurement taken if the first and second measurements do not agree with the pre-defined limits (Table 1).[9]

Weight will be measured using the SECA-876 scale with a mother-child-function (range=0-250kg, precision±0.1kg). Adult height (standing and sitting) and infant length will be measured using a portable UNICEF-height/length board (range=0-221cm, precision±0.1cm), and for sitting height using a purpose-built anthropometric box.[10] Mid-upper arm circumference (MUAC) and infant head circumference will be measured using the Lufkin-WP601 tape-measure. Both infant and adult arm measurements will be done on the left-hand side. Knee-to-heel length will be measured using a segmometer (Malby labs-India and Indonesia and Sealy-Senegal). Skinfolds measurements will be taken using a Holtain skinfold-caliper (range 0-40mm, precision±0.2mm).

Confidential: For Review Only

In the data cleaning process consistency checks in CommCare for infant z scores; length-for-age (<-6 or >6sd), weight-for-length (<-5 or >5sd) and weight-for-length (<-6 or >5sd),[9, 11] and for a negative difference between the current and preceding measurement of infant weight and length will be done. Z-scores will be calculated using the WHO child growth standards software.[12] Prior to data collection, in each country, a standardisation session will take place to calculate intra- and inter-observer absolute and relative technical error of measurements and coefficients of reliability for all anthropometric measurements.[9, 11] Regular refresher training sessions will be conducted and, field supervisors will monitor data collection, using a checklist based on the study's Standard Operating Procedures (SOP).

Biochemical assessment of blood samples

Using country-specific standard procedures,[13] blood samples will be collected from pregnant women (i.e., 10 mL in India and Senegal and 7 mL in Indonesia) during the third trimester (and second trimester in a sub-sample of women enrolled in a concurrent egg intervention trial in Indonesia). In Infants, 3 mL in India and Senegal and 2.5 mL in Indonesia of non-fasting blood samples will be collected at 6 months of age (and at 18-months in a sub-sample of children enrolled in an egg intervention study in India). The samples will be collected using Becton-Dickinson vacutainers®(EDTA and trace-element free). A complete blood count and HbA1C (women only) will be analysed on whole blood samples within 4-8 hours of collection. Blood samples will then be processed for separation of plasma, red blood cells and serum and aliquoted into microtubes for storage. All aliquots will be stored at -80°C until analyses (Table 2). The time of blood sample collection and separation will be recorded. The risks of contamination/analyte deterioration will be minimized by wearing powder-free gloves, keeping samples cool and protection from light.

Blood samples will be analysed for maternal and infant biochemical indicators of nutritional status, inflammation, and growth factors in infants (Supplementary Table 2). Certified reference materials (CRM) will be included in the analyses where available to ensure analytical accuracy, precision and reliability. A certified pooled plasma sample (Human Plasma Pool (H100500) from European Pharmacopoeia for Indonesia) will also be analysed across all laboratories to ensure inter-country comparability and analytical reliability.

Dietary intakes

Four-pass 24-hour dietary recall (MP24HR),[14] will be used to assess dietary intake. During the second (Indonesia and Senegal) and third trimesters (all sites), women will be asked to recall all foods and beverages consumed in a preceding 24 hours. The infants' dietary intakes from

Confidential: For Review Only

complementary foods will be estimated using and interactive 24-hour recall at 6, 9, 12, 18 and 24-months of age. For both mothers and children, a repeat recall will be collected on a randomly selected 10% sub-sample of the population to estimate intra-subject variability in nutrient intakes. The nutrient density of the complementary foods will be compared to a recommended nutrient density.[15] For children ≥12 months of age, breast milk intakes will be estimated as the difference between mean estimated energy requirements and the mean energy intakes from complementary foods to estimate total daily intakes of energy and nutrients. The percentage of mothers and 1-year old children at risk of inadequate nutrient intakes will be estimated, depending on the nutrient, using the Estimated Average Requirement fixed cut-point approach or the full probability approach Quantities of foods and drinks consumed will be estimated by weighing actual foods/beverages or food models using calibrated digital electronic kitchen scales (Salter, 1024,-India and Senegal and Tanita, KD-160-Indonesia; precision±1.0 g), dimension measurements (cm), size estimations (small, medium or large) or using photographs, depending on the item. Details on sources of food acquisition will be recorded, and questions will be asked about whether their food intake on the preceding 24-hours was typical of their usual intakes, and for infants, whether they were breastfed.

Local conversion factors to convert portion size estimates to grams consumed and average recipe data to convert the quantity of a mixed-dish consumed into grams of individual ingredients will be developed when they are not available from previous studies.

In Indonesia, MP24HR data will be entered on a tablet (CommCare-application),[16] whilst in India and Senegal, a paper form will be used to collect dietary data from respondents and entered on a web-based application created specifically for this project and then uploaded on to the study data repository. Both applications (paper and tablet-format) will calculate each participant’s intakes of energy and nutrients using the local conversion factors, average recipe data and country-specific food composition tables (FCT) in India and Indonesia,[16,17] and a FCT developed using a hierarchy of other food composition values in Senegal.[18–24] Paper-based forms will be reviewed immediately after data collection by the field supervisors and any questions resolved.

Ultrasound

Pregnant women in all three countries will have an antenatal ultrasound in government-recognised diagnostic centers at 32-weeks (± 2-weeks) to evaluate fetal growth and development.[25] A trained obstetric sonographer will use a ultrasound system,[26] to take the following measurements in triplicate, which will be checked by a radiologist; biparietal diameter, occipito-frontal diameter, head circumference, antero-posterior abdominal diameter, transverse abdominal diameter, abdominal circumference and femur length, fetal presentation, placental localisation and amniotic fluid volume,

Confidential: For Review Only

fetal movement, cardiac pulsation and the presence of congenital anomalies.[27] Estimated gestational age will be calculated using the crown-rump length.[28] The ultrasound report will be shared with the participant and advised to see the on-call doctor if necessary.

IYCF practices during the first 6 months after birth and breastmilk nutrient composition

Within 72-hours of birth, enumerators will administer a questionnaire to mothers to evaluate: 1)pre-lacteal feeding (other feeds, besides breastmilk), 2)breastfeeding initiation and timing, 3)colostrum fed to the infant, 4)other feeds, besides breastmilk, and 5)infant nutrition supplements.[29,30] Once a month until the infant is 6-months of age, the enumerator will ask mothers whether their infant has consumed anything other than breastmilk during the previous four-weeks. If the infant was not exclusively breastfed, detailed questions about the type of newly introduced liquids or solids will be asked.

A morning (between 6h00-12h00) breastmilk sample will be collected from mothers at 3-months post-partum by asking them to express all milk from one breast into a sterile collection bottle, using a manual breastmilk pump, following standard procedures.[31] After collection the bottles will be placed in an ice-box and transported to a laboratory, where milk samples will be mixed with a vortex-mixer and aliquot 1mL in amber 1.5mL tube (fatty acid, fat and water soluble vitamin analysis-Indonesia only), 10 mL into three amber-coloured screw cap 15mL Eppendorf-tubes® (fat- and water-soluble vitamins and fatty acids analyses for India and Senegal), one clear trace-element free tube (minerals analysis for India and Senegal) and 1 mL in clear 1.5 mL Eppendorf-tubes (oligosaccharides analysis for all three countries).[30] Samples will be immediately stored at -80°C (Table 2). CRM (NIST-1549a) – a standard control sample, will be included in the analyses to ensure analytical accuracy, precision and reliability.

Child morbidity

Child morbidity will be recorded using an adapted country-specific pictorial diary from birth to 24 months of age.[32] Mothers will be asked to complete the diary daily in India and Indonesia and collected weekly. Enumerators will help complete any incomplete forms. In Senegal, morbidity data will be collected weekly via enumerator-led telephone interviews. The same information will be collected across all three countries. Mothers will be asked if her child was healthy or had active, reduced appetite, diarrhoea (with or without blood and/or mucus in the stools), vomiting, bloated stomach, fever, cough (with or without nasal discharge and/or wheezing), ear pain, chicken pox or measles.[32] They will also record any health center visits and any medication the infant received.

Confidential: For Review Only

Additional morbidity questions (1-week recall) will also be asked at the same time as monthly IYCF questionnaire up to 6-months of age.

Maternal mental health and social support

Maternal stress will be assessed using cortisol level in hair samples,[33] and the Global Measure of Perceived Stress Scale (GMPSS) questionnaire.[34] Hair samples will be collected and GMPSS questionnaire administered during pregnancy (i.e., second trimester in Indonesia and Senegal and third trimester in India) and at 3 and 9 months post-partum. Hair grows approximately 1cm/month, therefore 3cm of hair will be collected as close as possible to the scalp from the posterior vertex of the head, placed on a hair collection card indicating the root end, wrapped in aluminum foil and stored in a dry, dark environment at room temperature until analysed, as described elsewhere.[33,35] Information on hair-care practices, which influence the hair-cortisol measurements, will also be recorded.[35] The GMPSS consists of 14 questions, on thoughts and feelings, which participants will rate using a 5-point Likert scale.[34]

Maternal depression will be measured using the Edinburgh Postnatal Depression Scale (EPDS) and the Self-Reporting Questionnaire (SRQ) during the second (Senegal and Indonesia) and third trimester (India), and then in all countries at 3, 9 and 18-months post-partum.[36,37] The EPDS consists of 10 statements, where participants are asked to rate how they felt in the last 7-days on a four-point Likert scale.[36] As well as the classic EPDS, the new shorter version of the EPDS will also be asked to validate it against the classic EPDS in India and Senegal.[38] The SRQ was developed by the WHO to screen for common mental disorders.[39] It consists of 20 questions with dichotomous yes/no answers.

Maternal social support will be assessed using the Multidimensional Scale of Perceived Social Support,[40] at 3, 9 and 18-months after birth. It includes 12 questions where participants are asked to rate how strongly they agree or disagree on a 7-point Likert scale,[40] and their responses are recorded.

Data collected by other workstreams within the AASH study

The Action Against Stunting Hub incorporates interdisciplinary workstreams and will be collecting data on the following; detailed household questionnaires, epigenetics (mother, father and infant saliva samples), gut health and the microbiome (mother and infant stool samples), Water, Sanitation and Hygiene in the home environment (household observations), food systems (market and household observations and surveys) and education and cognition (household and pre-school observations).

Confidential: For Review Only

Statistical analysis plan

Data will firstly be analysed at country level and secondly pooled together where appropriate. The primary outcomes are: 1) growth velocity between time points from birth to 18 months and 2) height for age z scores at timepoints 3, 6, 9, 12 and 18 months. Based on the literature, we have decided a priori to include the following parental covariates in our models; age, cast (India only), village/health post, socioeconomic status, marital status, current work status and education status. Multivariable statistical models will be developed using Directed Acyclic Graphs and related approaches including structural equation modelling with pre-analysis to understand confounders and mediators on the pathway between exposures and outcomes. We will build our models and adjust for confounders using a forward stepwise approach with Akaike Information Criterion to identify the model of best fit.[41] All analysis will be conducted using STATA version 17 and R.

Patient and public partnership statement

Patients and public were not involved in the design of this study

Discussion

Addressing early life undernutrition is a public health priority in LMICs given its detrimental impacts on child survival, growth and development. The full extent and aetiology of undernutrition, however, are not well established. given the limited number of cohort studies from fetal life through to early childhood. As such studies are needed to understand the complex interplay among environmental and intrinsic physiological factors influencing early life nutritional status in different contexts.

This study and others within the Action Against Stunting Hub (AASH) will investigate these complex relationships across three distinct geographies in sub-Saharan Africa and Asia. The analysis will examine factors affecting infant growth through three distinct periods; fetal period, 0-6 months of age and during the complementary feeding (6-24 months of age). The selection of its data collection protocols is underpinned by a theoretical framework of factors influencing infant and childhood growth (Figure 1), which will be further expanded through AASH inter-disciplinary collaborations to include influences of epigenetics, gut-health and food-systems. This study will also contribute to less-researched areas such as the influence of maternal depression, maternal stress and social support on infant care and feeding practices which ultimately affect infant-growth.

There are several limitations to this study. Firstly, it was not feasible, for this cohort study to include the preconception period given the time frame and resources available. Also, recruitment in the first trimester would not have been appropriate given cultural sensitivities, in some participating countries,

Confidential: For Review Only

of admitting pregnancy in the first trimester. The collection of data during the 2nd and 3rd trimester of pregnancy, however, will provide a wealth of relevant data related to exposures during pregnancy that will influence fetal and later infant growth, including a measure of weight gain in pregnancy, stress (hair cortisol reflects stress for the past 3 months) and maternal dietary intakes. Secondly, although identical indicators of micronutrient status will be analysed across countries, the methods of analyses for two biochemical indicators differ. To minimize the influence of inter-laboratory assay differences on cross-country comparisons, a pooled-sample and CRMs, where available, will be analysed in all laboratories. Thirdly, in our cohort, since we enrolled the participants in the second (Indonesia and Senegal) and the third trimester (India) of pregnancy, the study teams could only carry out ultrasound scans during at these time-points rather than the first trimester. However, data from the first and second trimester scans are available in around 80% and 90% participants in India, respectively and will be utilized for gestational age estimation if available. If not, the date of last menstruation will be used. Studies have shown that routine ultrasound scans at around 32 weeks will help in detection of early Intrauterine growth restriction (IUGR).[42] Therefore, within our limitations to perform the ultrasound scan at 2nd and third trimesters as a part of the study protocol, we think these ultrasound measurements will be useful to identify early IUGR by comparison with INTERGROWTH-21 charts. This information, coupled with the infant growth data will be valuable because such data are scarce in low resource setting.[43]

Fourthly, inter-population differences in maternal literacy necessitated a change in morbidity data collection protocols in Senegal. Lastly, when investigating causal inferences, there are limitations with a prospective cohort study design, including loss-to-follow-up bias and confounding. These biases will be minimised through concerted sensitisation/motivational efforts to reduce loss-to-follow-up and the use of DAGS.

Conclusions

Results from the nutrition study conducted in three diverse geographic regions will form part of the ‘whole child approach’ pursued under AASH. The interdisciplinary collaborations established across the hub provide a unique opportunity to investigate the complex inter-relationships among environmental and intrinsic physiological factors influencing early life growth and development. Such insights are important for informing effective intervention design and the policy actions needed to support global efforts in reducing early child-stunting.

https://mc.manuscriptcentral.com/bmjpo

Table 1: Pre-defined limits in the difference between first and second anthropometry measurements

Measure	Pre-defined limits
Adult and infant weight	0.1 kg
Adult height	0.5 cm
Adult Mid Upper Arm Circumference	0.5 cm
Adult skinfolds	0.2 cm/2 mm
Infant length	0.7 cm
Infant Head circumference	0.5 cm
Infant knee to heel	0.5 cm
Infant Mid Upper Arm Circumference	0.5 cm
Infant skinfolds	0.2 cm/2 mm

Confidential: For Review Only

Table 2: Analysis methods for parameters in India, Indonesia and Senegal

**Parameters**	**India**	**Indonesia**	**Senegal**
**Blood samples**			
Complete Blood Count<br>Glycated Haemaglobin (HbA1C -pregnant women only)	CBC auto-analyser (Horiba ABX Micros ES60)<br>HbA1C analyser (Affinion ™ Hb1Ac, Abbot)	CBC auto-analyser (SYSMEX XP100)<br>HbA1C analyser (PocketChem A1c, Arkray Inc)	CBC auto-analyser<br>HbA1C analyser (HPLC D10 Biorad)
Red Blood Cell folate<br>Methylmalonic acid (MMA)<br>Choline<br>Betaine	LC-MS/MS[1]	LC-MS/MS	LC-MS/MS
Homocysteine<br>Vitamin D<br>Vitamin B12<br>Vitamin B9	LC-MS/MS	LC-MS/MS	CLIA[2]
Red Blood Cell-Fatty acids	GC-FID[3]	GC-FID	GC-FID
Amino Acids	HPLC	LC-MS/MS	LC-MS/MS
Retinol	HPLC	HPLC	HPLC
Ferritin<br>TfR	ELISA[4]	ELISA-Quansys multiplex system	CLIA<br>ELISA
C-reactive protein<br>α1-Acid glycoprotein<br>Hepcidin<br>Retinol binding protein<br>Insulin like growth factor-1<br>Insulin-like growth factor binding protein<br>Intestinal fatty-acid binding protein	ELISA	ELISA-Quansys multiplex system	ELISA-Quansys multiplex system
Minerals/trace elements	ICP-MS[5]	ICP-MS	ICP-MS
Remaining Red blood cells<br>Remaining plasma	Kept at -80°C	Kept at -80°C	Kept at -80°C
**Breastmilk samples**			
Fat-soluble vitamins A and D	HPLC/LC-MS[6]	HPLC/LC-MS	HPLC/LC-MS
Water-soluble vitamins B1, B2, B3, B6, B9 and B12	LC-MS[7]	LC-MS/MS	LC-MS
Fatty acids	GC-FID	GC-FID	GC-FID
Minerals	ICP-MS	ICP-MS	ICP-MS
Oligosaccharides	SPEC[8] and HPLC	LC-MS/MS	SPEC and HPLC

Confidential: For Review Only

Remaining breastmilk	Kept at -80°C	Kept at -80°C	Kept at -80°C
**Hair samples**			
Cortisol	ELISA	LC-MS/MS	ELISA

[1] Liquid Chromatography with tandem Mass Spectrometry (LC-MS/MS), [2] Chemiluminescence Immunoassay, [3]High Performance Liquid Chromatography, [4] Gas Chromatography-Flame-ionisation detection (GC-FID), [5] Enzyme-Linked Immunoassay, [6]Inductively coupled plasma mass spectrometry, [7] High Performance Liquid Chromatography/ Liquid Chromatography-Mass Spectrometry, [8]Liquid Chromatography-Mass Spectrometry, [9] Solid Phase Extraction Chromatography

Confidential: For Review Only

References

1. Lee RD, Nieman DC. Nutritional Assessment. International Edition. In: Forth Edition. New York: McGraw-Hill; 2007.

2. Zoleko-Manego R, Mischlinger J, Dejon-Agobé JC, et al. Birth weight, growth, nutritional status and mortality of infants from Lambaréné and Fougamou in Gabon in their first year of life PLOS ONE. 2021;16(2).

3. Woldeamanuel GG, Geta TG, Mohammed TP, Shuba MB, Bafa TA. Effect of nutritional status of pregnant women on birth weight of newborns at Butajira Referral Hospital, Butajira, Ethiopia. SAGE Open Med. 2019;7.

4. Black R, Allen L, Bhutta Z, Caulfield LE, de Onis M, Ezzati M. Maternal and child undernutrition: global and regional exposures and health consequences. Lancet. 2008;371(9608):243–60.

5. UNICEF. Nutrition, for every child UNICEF Nutrition Strategy 2020-2030. UNICEF Nutrition Section, Programme Division; 2020.

6. National Agency for Statistics and Demography - ANSD / Senegal and ICF. Demographic and Continuous Health Survey (EDS-Continue) 2017 [Internet]. Dakar, Senegal: ANSD and ICF; 2018. Available from: http://dhsprogram.com/pubs/pdf/FR345/FR345.pdf.

7. International Institute for Population Sciences. National Family Health Survey NFHS-4, 2015-16. India, Mumbai:IIPS; 2017.

8. Agency of Health Research and Development. Indonesia Basic Health Research 2018 . Indonesia; 2018.

9. de Onis M, et al. Measurement and standardization protocols for anthropometry used in the construction of a new international growth reference. Food Nutr Bullitin. 2004;25(S1):S27–36.

10. Norton K. International Standards for Anthropometric Assessment. International Society for the Advancement of Kinanthropometry; 2001.

11. WHO. RECOMMENDATIONS FOR DATA COLLECTION, ANALYSIS AND REPORTING ON ANTHROPOMETRIC INDICATORS IN CHILDREN UNDER 5 YEARS OLD. Geneva: World Health Organization and the United Nations Children's Fund (UNICEF); 2019. Report No.: Licence: CC BY-NC-SA 3.0 IGO.

12. WHO: Geneva. WHO Anthro for personal computers, version 3.2.2, 2011: Software for assessing growth and development of the world's children. [Internet]. 2010. Available from: (http://www.who.int/childgrowth/software/en/

13. Centre for Disease Control and Prevention. National Health and Nutrition Examination Survey (NHANES) MEC Laboratory Procedures Manual. 2020.

14. Gibson RS, Ferguson EL. An Interactive 24-Hour Recall for Assessing the Adequacy of Iron and Zinc Intakes in Developing Countries. Washington, DC, USA: International Life Sciences Institute; 2008.

15. Dewey KG, Brown K. Update on technical issues concerning complementary feeding of young children in developing countries and implications for intervention programs Food Nutr Bull. 2003;24(2):239.

16. Htet MK, Fahmida U, Do TT, Dibley MJ, Ferguson E. The used of tablet-based multiple-pass 24-hour dietary recall application (MP24Diet) to collect intake of children under two years old in the prospective cohort study in Indonesia. Nutrients. 2019;11(12):2889.

17. Data Komposisi Pangan Indonesia (Indonesian Food Composition Table). https://www.panganku.org/id-ID/beranda. 2018.

Confidential: For Review Only

18. Longvah T, Anathan R, Bhaskarachary K, Venkalah K. Indian Food Composition Tables. National Institute of Nutrition, Indian Council of Medical Research, Department of Health Research, Hyderabad, India; 2017.

19. FAO/INFOODS. Food Composition Table for Western Africa (2019) /Table de composition des aliments FAO/INFOODS pour l'Afrique de l'Ouest. 2019.

20. Nigeria Food Database [Internet]. Available from: http://nigeriafooddata.ui.edu.ng/

21. Prynne C, Paul A. Food Composition Table for Use in The Gambia. Cambridge: Medial Research Council Human Nutrition Research; 2011.

22. SAFOODS, SA MRC. South African Food Data System [Internet]. 2018. Available from: http://safoods.mrc.ac.za/

23. Public Health England. McCance and Widdowson's The Composition of Foods Integrated Dataset 2021. 2021. Report No.: GW-2010.

24. US Department of Agriculture. Food and Nutrition Database [Internet]. Available from: https://fdc.nal.usda.gov/

25. Salomon L, Alfirevic Z, Da Silva-Costa F. ISUOG Practice Guidelines: ultrasound assessment of fetal biometry and growth. Ultrasound Obstet Gynecol. 2015;46(4):391–7.

26. Phillips Affiniti 50 Ultrasound system [Internet]. Available from: https://www.philips.co.in/healthcare/product/HC795208/affiniti-50-ultrasound-system

27. Romosan G, Henriksson E, Rylander A, Valentin L. Diagnostic performance of routine ultrasound screening for fetal abnormalities in an unselected Swedish population in 2000–2005. Ultrasound Obstet Gynecol. 2009;34(5):526–33.

28. Gadsbøll, K, Wright A, Kristensen SE, et al. Crown–rump length measurement errors: impact on assessment of growth. Ultrasound Obstet Gynecol. 2021;58(3):354–9.

29. Caulfield LE, Bose A, Chandyo RK, , et al. Infant Feeding Practices, Dietary Adequacy, and Micronutrient Status Measures in the MAL-ED Study. Clin Infect Dis. 2014;59 Suppl 4:S248-54.

30. WHO. Indicators for assessing infant and young child feeding practices. Part 2 Measurement. Geneva, Switzerland; 2010.

31. Leghi GE, Middleton PF, Netting MJ, Wlodek ME, Geddes DT, Muhlhausler BS. A Systematic Review of Collection and Analysis of Human Milk for Macronutrient Composition. Methodol Math Model. 2020;00:1–19.

32. Richard SA, Barrett L, Guerrant R. Disease surveillance methods used in the 8-stie MAL-ED cohort study. Clin Infect Dis. 2014;59(Suppl 4):S220–4.

33. Greef M, Levine J, Abuzgaia A, Elzagallaai A. Hair cortisol analysis: An update on methodological considerations and clinical applications. Clin Biochem. 2019;63:1–9.

34. Cohen S, Kamarck T, Mermelstein R. A global measure of perceived stress. J Health Soc Behav. 1983;24:385–96.

35. Wright K, Ford J, Perazzo J, et al. Collecting Hair Samples for Hair Cortisol Analysis in African Americans. J Vis Exp. 2018;136(e57288):1–7.

36. Cox JL, Holden JM, Segovsky R. Edinburgh Postnatal Depression Scale (EPDS). Br J Psychiatry. 1987;150.

37. Harding T, DeArango V, Baltazar J. Mental disorders in primary health care: a study of their frequency and diagnosis in four developing countries. Psychol Med. 1980;10:231–41.

Confidential: For Review Only

38. van Heyningen T, Myer L, Tomlinson M, Honikman S. The development of an ultra-short, maternal mental health screening tool in South Africa. Glob Ment Health. 2019;6(e24):1–14.

39. Beusenberg M, Orley JH. A User's guide to the self reporting questionnaire: A User's guide to the self reporting questionnaire [Internet]. Geneva: World Health Organisation. Division of Mental Health; 1984. Available from: https://apps.who.int/iris/handle/10665/61113

40. Zimet GD, Dahlem NW, Zimet SG, Farley GK. The Multidimensional Scale of Perceived Social Support. J Pers Assess. 1988;52:30–41.

41. Kirkwood B, Sterne J. Essential Medical Statistics. 2nd ed. Blackwell Science: Oxford; 2003.

42. Sovio U, White I, Darcey A, Pasupathy D. Screening for fetal growth restriction with universal third trimester ultrasonography in nulliparous women in the Pregnancy Out-come Prediction (POP) study: a prospective cohort study. Lancet. 2015;21;386(10008):2089–97.

43. Gordijn, Beune I, Thilaganathan B, Papageorghiou A, Baschat A, Baker P. Consensus definition of fetal growth restriction: a Delphi procedure. Ultrasound. Ultrasound Obstet Gynecol. 2016;3(48):333–9.

Confidential: For Review Only

Authors' contributions: HDK and ELF were responsible for the overall design, training and overseeing implementation of the research. UF, MKH, BK, BF, RM, RP, RP, TR, SFR, SD, RP, SA, AW, JW, PH, CH were involved in its design. UF, MKH, BK, BF, DY, DS, NLZ, TCA, RM, RP, RP, TR, SFR, SKB KS, DP, , DY, SD, PL-S, BD, PM, SF, ID, AD, TVI, FT, AD, SS, BMK, DTT implemented the research. HDK and ELF wrote the manuscript. All authors read, provided comments on and approved the final version of the manuscript.

Funding: This study is funded by UKRI Global Challenges Research Fund (GCRF) (No. MR/S01313X/1). The views expressed in this work are those of the authors and not the funder.

Competing interest statement: The authors declare no conflict of interest.

Confidential: For Review Only

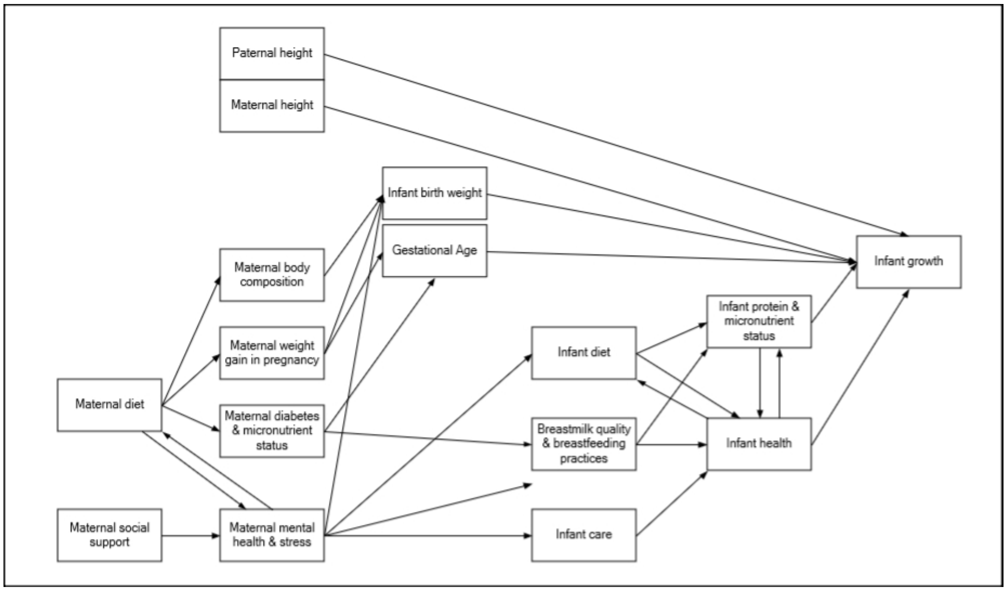

Figure 1_A conceptual framework of pathways to assess the impact of nutrition nutritional status and maternal wellbeing on child stunting

244x142mm (96 x 96 DPI)

Supplementary Table 1: Flow chart for data collection timing

Measurements	2nd trim[1]	3rd trim	Birth	1-2 mo[2]	3 mo	4-5 mo[2]	6 mo	9 mo	12 mo	18 mo	24 mo
**Anthropometry**											
Pregnant women	IS[3]	All[4]									
Women weight only				All	All	All	All	All	All	All	All
Biological fathers			All								
Infants			All		All		All	All	All	All	All
Infants weight only				All		All					
**Blood Sample**											
Pregnant women	IS	I[5]									
Infants							All				
**Dietary Intakes**											
Pregnant women	IS	All									
Infants							All	All	All	All	All
**Ultrasound**		All									
Pregnant women											
**Breastfeeding**											
Breast milk sample					All						
Questionnaires			All	All	All	All	All				
**Morbidity[6]**			All	All	All	All	All	All	All	All	All
**Mental Health**											
Hair cortisol	IS	I			All						
GMPSS[7]	IS	I			All			All		All	
EPDS[8]	IS	I			All			All		All	
MSPSS[9]	IS	I			All			All		All	
SRQ[10]		All			All			All			

Confidential: For Review Only

[1]–trimester; [2] – data collected each month; [3] – Indonesia and Senegal only; [4] – all three countries; [5] - India only; [6] – infant morbidity data is recorded everyday; [7] - Global Measure of Perceived Stress Scale (GMPSS); [8] - Edinburgh Postnatal Depression Scale (EPDS); [9] - Multidimensional Scale of Perceived Social Support (MSPSS), [10] Self-Reporting Questionnaire (SRQ)

Confidential: For Review Only

Supplementary Table 2: Summary of the data that will be collected from each participant and the main indicators generated for statistical analysis.

Data collection	Main Indicators
**Anthropometry**	
Pregnant women: Weight, height, sitting height, mid-upper-arm-circumference (MUAC) and skinfolds (triceps, biceps, sub-scapular)	***Pregnant women***: Weight gain in pregnancy [1]**(con)**, sitting height to standing height ratio**(con)** and arm muscle and fat areas **(con)**
Women post-partum: Weight	***Maternal:*** Post-partum body-mass index (BMI) [2]**(cat)** and % under-weight/over-weight /obese **(con)**
Biological father: Weight, height, sitting height, MUAC and skinfolds (triceps, biceps, sub-scapular for India and Senegal) and body composition for Indonesia.	***Paternal:*** BMI **(cat)**, % under-weight/over-weight /obese **(con)**, sitting height to standing height ratio **(con)**, and arm muscle and arm fat areas **(con)**
Infant: Birth weight, birth length, head circumference, knee-to-heel length, weight, length, MUAC, skinfolds (triceps, sub-scapular)	***Infant***: Birth weight **(con)**, % low birth weight **(con)**, Z-scores[3] for weight-for-age **(cat)**, weight-for-length **(cat)**, length-for-age **(primary outcome-cat)**, length **(primary outcome-con)**, BMI **(cat)**, skinfolds **(con)**, arm and head circumferences **(con)**, Z-scores[3] for velocity-for-length **(primary outcome-con)**, weight and head circumference **(con)**, Z-score for weight-for-length[3] **(con), %** stunted **(con)**, % wasted **(con)** and % low arm circumference **(con).**
**Blood Sample**	

Confidential: For Review Only

Pregnant women: Blood sample in (1) BD EDTA lavender tube (6 ml India and Senegal and 5.75 ml Indonesia); (2) BD royal blue top trace element free tube (4 ml India, 1 ml Indonesia and 5 ml Senegal); and (3) BD microtainer (120 µl India and Senegal and 250 µl Indonesia)<br><br>***Infants:*** Non-fasting blood sample in (1) BD EDTA lavender tube (2.5 ml India and Senegal and 1.5 ml Indonesia); (2) BD royal blue top trace element free tube (0.7 ml India, 1 ml Indonesia and 2.5ml Senegal); and (3) BD microtainer (20 µl India, and 40 µl Senegal)	***Pregnant women*:** (1) Micronutrient status (RBC folate, Plasma folate**,** B vitamins, homocysteine, methylmalonic acid, choline, betaine, complete blood count, ferritin, transferrin receptor, hepcidin, retinol binding protein, retinol, amino acids, Fe, Zn, Cu, Se, Ca, Cr, Cd, Co, Pb, Hg, As, Al, and 25-dihydroxyvitamin D) **(con)**; (2) RBC-fatty acids **(con)**; (3) Gestational Diabetes with HbA1C **(cat)**; and (4) Inflammation with C-reactive protein, α1-acid glycoprotein **(con)**<br><br>***Infants***: Micronutrient status (Folate, B vitamins, methylmalonic acid, choline, betaine, complete blood count, ferritin, transferrin receptor, hepcidin, retinol binding protein, retinol, RBC-fatty acids and Folate, Fe, Zn, Cu, Se, Ca, Cr, Cd, Co, Pb, Hg, As, Al) **(con);** (2) RBC-fatty acids **(con)**; (3) Amino acids **(con)**; (4) Growth Hormone, Insulin-like growth factor-1, IGF binding protein, intestinal fatty acid binding protein **(con)**, and (5) Inflammation with C-reactive protein, α1-acid glycoprotein **(con)**
**Dietary Intakes**<br><br>***Pregnant women:*** Multiple pass 24-hour diet recall with repeat in 10% of women at each time point<br><br>***Infants:*** Multiple pass 24-hour diet recall with repeat in 10% of infants at each time point	***Pregnant women:*** Intakes of selected foods/beverages (g/d) **(con)**, energy and nutrients **(con)**, % at risk of inadequate nutrient intakes **(con)**, % energy from selected food groups and food sub-groups **(con)**<br><br>***Infants:*** Intakes of selected foods/beverages (g/d), energy and nutrients **(con)**, the nutrient density of complementary feeding diets in relation to desired levels **(con)**, % energy from selected food groups and food sub-groups **(con)**, % continued breastfed to 24-months of age **(con),** complementary feeding (introduction of solid, semi-solid

Confidential: For Review Only

	or soft foods 6–8 months, minimum dietary diversity 6–23 months, minimum meal frequency 6–23 months, minimum milk feeding frequency for non-breastfed children 6–23 months, minimum acceptable diet 6–23 months, egg and/or flesh food consumption 6–23 months, sweet beverage consumption 6–23 months, unhealthy food consumption 6–23 months and zero vegetable or fruit consumption 6–23 months)<br><br>***Infants ≥12-months:*** % at risk of inadequate nutrient intakes and nutrient density **(con)**
**Ultrasound**<br><br>**Pregnant woman and developing infant:** 1) Biparietal diameter, 2) occipito-frontal diameter, 3) head circumference, 4) antero-posterior abdominal diameter, 5) transverse abdominal diameter, 6) abdominal circumference (AC) and 7) femur length, 8) fetal presentation, 9) placental localization, 10) amniotic fluid volume, 11) fetal movement, 12) cardiac pulsation, 13) presence of congenital anomalies and 14) gestational age (GA)	 <br>**Pregnant woman and developing infant:** Intrauterine growth retardation (IUGR), gestational age **(con)** and fetal-weight-for-gestational age **(con)**
**Breastfeeding**	

Confidential: For Review Only

Breast milk sample: Full expression of a morning breastmilk sample from one breast<br><br>***Questionnaire at birth***: Administered within 72 hours of birth on initial breastfeeding practices<br><br>***Questionnaire in first 6-months***: Administered once per month on breastfeeding practices	***Breastmilk sample:*** the breastmilk content of fatty acids, fat- and water-soluble vitamins, minerals and oligosaccharides **(con)**<br><br>***Questionnaire at birth***: % ever breastfed **(con)**, % early initiation of breastfeeding **(con)**, % pre-lacteal feeding **(con)**<br><br>***Questionnaire in first 6-months***: % exclusively breastfed **(con)**, % mixed milk feeding **(con)**, age of introduction of semi-solid or solid foods **(con)**
**Infant Morbidity**<br><br>***Paper based pictorial chart:*** Completed daily by caregivers on infant health status, illness, clinic visits and medication use	***Paper based pictorial chart:*** % frequency and number of days of reduced appetite, stomach pain[4], bloated stomach[4], diarrhoea, vomiting fever, cough, wheezing, nasal discharge, ear pain[5], chicken pox[5] or measles[5], health center visits and prescribed medication **(con)**
**Maternal mental health& well-being**<br><br>***Hair samples:*** Collected from the posterior vertex of the head from pregnant women ***Questionnaires***: Administered to the woman during pregnancy and post-partum	Hair cortisol content as an indicator of stress (>182 pg/mg) **(con)**

Confidential: For Review Only

	Global Measure of Perceived Stress Scale (GMPSS) (0-13=low perceived stress, 14-26=moderate perceived stress and 27-40=high perceived stress) **(cat)**<br><br>Edinburgh Postnatal Depression Scale (EPDS) cut-off of 13 **(cat)**<br><br>Multidimensional Scale of Perceived Social Support (MSPSS) (12-35 indicates low perceived support, 36-60 indicates medium perceived support and 61-84 indicates high perceived support) **(cat)**

[1]continuous **(con)**; [2]categorical **(cat)**

[3]Z-scores will be calculated utilizing the WHO child growth standards software (12), [4]Senegal only, [5]India and Indonesia only,

Confidential: For Review Only
